# Somatostatin-Expressing Neurons in the Ventral Tegmental Area Innervate Specific Forebrain Regions and Are Involved in Stress Response

**DOI:** 10.1523/ENEURO.0149-23.2023

**Published:** 2023-08-28

**Authors:** Elina Nagaeva, Annika Schäfer, Anni-Maija Linden, Lauri V. Elsilä, Ksenia Egorova, Juzoh Umemori, Maria Ryazantseva, Esa R. Korpi

**Affiliations:** 1 Department of Pharmacology, Faculty of Medicine, University of Helsinki, 00014 Helsinki, Finland; 2 Gene and Cell Technology, A. I. Virtanen Institute for Molecular Science, University of Eastern Finland, 70210 Kuopio, Finland; 3 HiLIFE Neuroscience Center, University of Helsinki, 00014 Helsinki, Finland

**Keywords:** behavioral phenotypes, midbrain, opioid sensitization, somatostatin neurons, stress reactions, VTA projection neurons

## Abstract

Expanding knowledge about the cellular composition of subcortical brain regions demonstrates large heterogeneity and differences from the cortical architecture. Previously we described three subtypes of somatostatin-expressing (Sst) neurons in the mouse ventral tegmental area (VTA) and showed their local inhibitory action on the neighboring dopaminergic neurons (Nagaeva et al., 2020). Here, we report that Sst+ neurons especially from the anterolateral part of the mouse VTA also project far outside the VTA and innervate forebrain regions that are mainly involved in the regulation of emotional behavior, including the ventral pallidum, lateral hypothalamus, the medial part of the central amygdala, anterolateral division of the bed nucleus of stria terminalis, and paraventricular thalamic nucleus. Deletion of these VTA^Sst^ neurons in mice affected several behaviors, such as home cage activity, sensitization of locomotor activity to morphine, fear conditioning responses, and reactions to the inescapable stress of forced swimming, often in a sex-dependent manner. Together, these data demonstrate that VTA^Sst^ neurons have selective projection targets distinct from the main targets of VTA dopamine neurons. VTA^Sst^ neurons are involved in the regulation of behaviors primarily associated with the stress response, making them a relevant addition to the efferent VTA pathways and stress-related neuronal network.

## Visual Abstract

**Figure:**
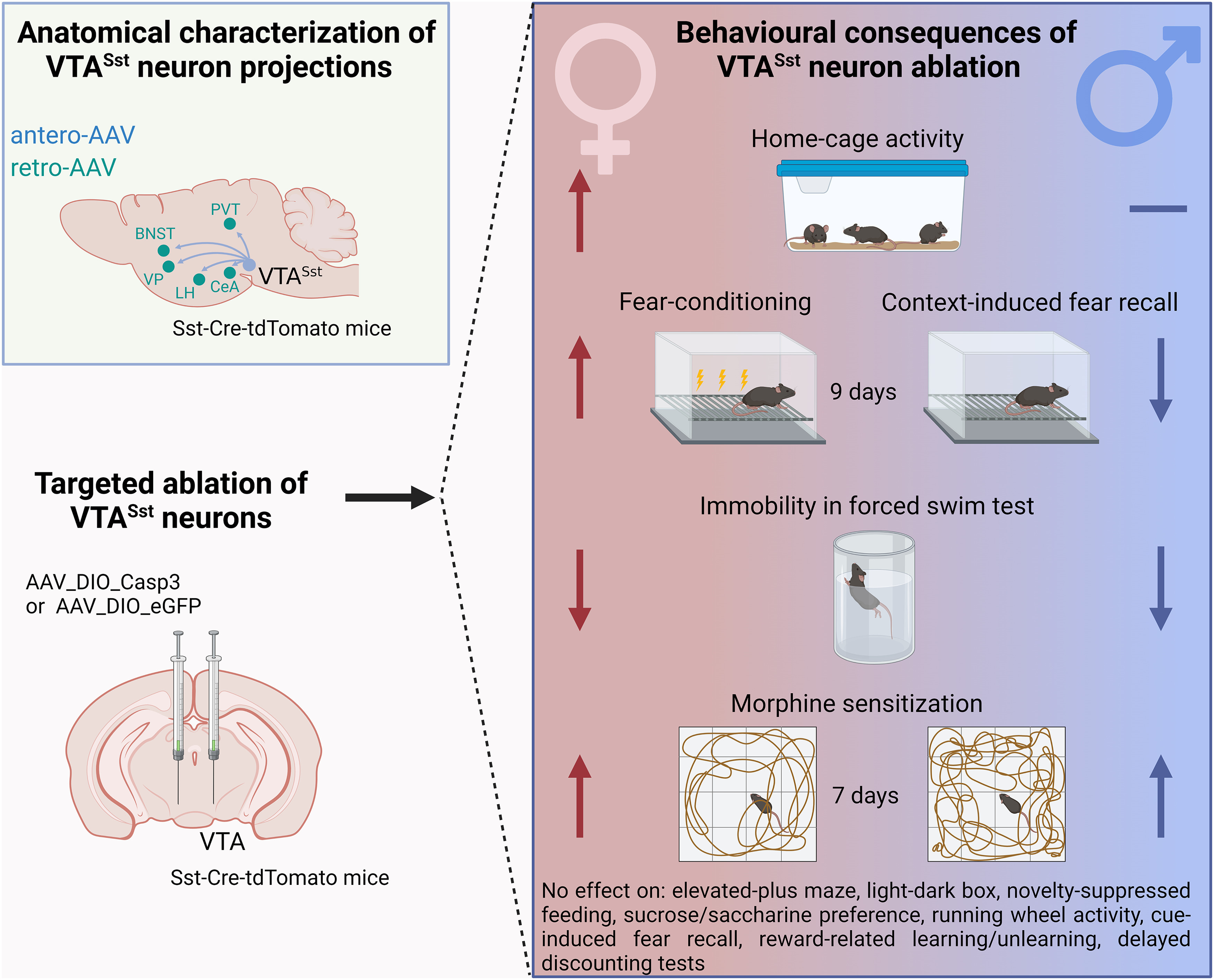

## Significance Statement

The VTA is involved in many reward- and aversion-related processes contributing to brain disorders, but the cellular composition and brainwide connections of this subcortical brain region are still poorly known. We previously described a heterogeneous Sst neuron population in the mouse VTA, and, here, we reveal that in addition to being local interneurons, VTA Sst neurons send long-distance projections to specific forebrain regions. Deletion of these neurons altered behavioral reactions to acute stressors and to repeated morphine administration. The results add the VTA Sst neurons to the stress-related network of the brain and establish a possibility to find out the exact roles of these neurons acting as behaviorally relevant interneurons and/or projection neurons.

## Introduction

The ventral tegmental area (VTA) is a part of the midbrain from which it sends neuronal projections to many brain structures. It is mainly recognized as the origin of two important dopaminergic pathways, the mesolimbic pathway to the ventral striatum and the mesocortical pathway to the prefrontal cortex, which control motivation and reward-related processes (Björklund and Dunnett, 2007). However, the VTA additionally contains two other major neuronal subtypes, neurons releasing glutamate (Glu) and GABA. In addition to participating in local circuits and controlling the neighboring dopamine (DA) cells (Dobi et al., 2010; Hnasko et al., 2012; Tan et al., 2012), some of these neurons also project outside the VTA and contribute to larger brain circuits. VTA GABA neurons can project to distant brain areas and modulate the activity of those areas separately from DA signaling by having unique projection targets (Bouarab et al., 2019). For example, continuous activation of inhibitory projections from the VTA to the epithalamic lateral habenula promotes rewarding behaviors independently of DA neurons (Stamatakis et al., 2013). At the same time, activation of the rostral VTA GABA neurons projecting to the GABA neurons in the dorsal raphe disinhibits serotonin neurons and promotes aversion (Li et al., 2019).

Expression of nonoverlapping marker genes by neuronal populations has extensively been used in neuroscience research and especially for the description of diverse GABAergic populations (Rudy et al., 2011; Tasic et al., 2018). Neuropeptide somatostatin (SST) is one of the major markers for cortical GABAergic neurons, along with Ca^2+^-binding protein parvalbumin (PV) and ionotropic 5-HT_3a_ receptor (Rudy et al., 2011). Although these three proteins have distinct functions within the cell, researchers found it convenient to use their genes for separating neuronal populations and developed mouse lines for their specific manipulation (Hippenmeyer et al., 2005; Taniguchi et al., 2011). Using a Sst-Cre mouse line, we described three Sst+ neuron populations in the VTA (VTA^Sst^) with heterogeneous molecular profile (75% GABA, 18% Glu, and 5% GABA/Glu) locations within the VTA and electrophysiological properties (Nagaeva et al., 2020). We also demonstrated that laterally located VTA^Sst^ neurons were able to inhibit neighboring DA cells via direct GABAergic transmission.

Given the increasing number of publications reporting the ability of Sst+ neurons to innervate distant areas in the brain and modulate a variety of behaviors in other subcortical regions, for example, in the amygdala (McDonald and Zaric, 2015), bed nucleus of the stria terminalis (BNST; Magableh and Lundy, 2014; Xiao et al., 2021), and lateral hypothalamus (LH; Luo et al., 2018), we questioned whether VTA^Sst^ neurons possess similar properties. Our findings demonstrate that anterolateral VTA^Sst^ neurons can indeed project to distant forebrain regions. Further, we performed a comprehensive screening for the possible behavioral consequences of deleting this VTA neuron population and observed that several stress responses were affected, often in a sex-dependent manner. Additionally, our data suggest that the majority of the projecting VTA^Sst^ neurons belong to the most abundant electrophysiological subtype of Sst+ neurons—the afterdepolarizing neurons (Nagaeva et al., 2020).

## Materials and Methods

### Animals

All experimental procedures were performed on male and female mice of heterozygous Sst-IRES-Cre (Sst^tm2.1(cre)Zjh^/J) genotype resulted from cross-breeding of Sst-IRES-Cre (Sst^tm2.1(cre)Zjh^/J) strain with Ai14 tdTomato reporter strain (B6.Cg-Gt(ROSA)26Sor^tm14(CAG-tdTomato)Hze^/J). Only the backtracing part was performed in homozygous Sst-IRES-Cre mice to prevent leakage of tdTomato signal into the GFP-channel, complicating the detection of the backtraced Sst+ eGFP-positive neurons. Animals were group housed in individually ventilated (IVC) cages (GM500, Tecniplast Green Line) under a 12 h light/dark cycle (lights on from 6:00 to 18:00) with *ad libitum* access to food (Global Diet 2916C, pellet 12 mm; Envigo) and water, unless otherwise indicated. Cages were equipped with bedding (500 ml aspen chips, 5 × 5 × 1 mm, catalog #HP10KG, Tapvei), nesting material (aspen strips, catalog #PM90L, Tapvei), and a clear handling tube (10 cm length, 5 cm diameter). Animal experiments were authorized by the National Animal Experiment Board in Finland (Eläinkoelautakunta, ELLA; license numbers ESAVI/1172/04.10.07/2018 and ESAVI/1218/2021).

### Tracing

The mice were anesthetized with a mixture of isoflurane (4% for induction, 0.5–2% for maintenance; Vetflurane, Virbac) and air (flow rate 0.8–1 L/min), after which they were placed into a stereotaxic frame (Kopf Instruments). Before opening the incision on the scalp, iodopovidone was applied to the surgical region, and lubricative eye gel was applied to prevent corneal damage.

For the anterograde tracing studies, stereotaxic coordinates AP −3.3, ML ±0.3, DV −4.5 mm relative to bregma were used for the VTA, based on the mouse brain atlas (Franklin and Paxinos, 2007) and verified with dye injections. A unilateral injection of 200 nl of adeno-associated virus (AAV)2/8-Cag-Flex-Myr-eGFP viral construct (4 × 10^12^ genome copies/ml, construct-146-aav2-8, Neurophotonics.ca. https://tools.neurophotonics.ca/product/aav-cag-flex-myr-gfp/), was made with a flow rate of 0.1 μl/min. A precision pump system (KD Scientific) was used to control the injection rate.

For the retrograde tracing, the following stereotaxic coordinates were used (mm relative to bregma): for the anterolateral BNST (alBNST), AP −0.1, ML ±0.9, DV −4.2; for the medial part of central amygdala (CeM), AP −1.5, ML ±2.6, DV, −4.7; for the LH, AP, −1.4, ML ±1.1, DV, −5.3; for the paraventricular thalamic nucleus (PVT), AP −1.5, ML ±0.1, DV −3.2; and for the ventral palladium (VP), AP +0.6, ML ±1.3, DV −5.4. For each animal, a 600 nl unilateral injection of 1:1 mixture of green RetroBeads [1:10 dilution in distilled (d)H_2_O, Lumafluor] and pAAV2-hSyn-DIO-eGFP retrograde virus construct (7.6 × 10^12^ genome copies/ml; catalog #50457-AAVrg, Addgene) was made with a flow rate of 1.0 μl/min. RetroBeads were used only for marking the injection site and were not considered in the backtracing analysis. After the surgeries, the mice were administered 5 mg/kg carprofen subcutaneously (Norocarp Vet, 50 mg/ml; Norbrook Laboratories) for postoperative analgesia and were left to recover from anesthesia in a 37°C warming chamber until ambulatory. A recovery period of at least 3 weeks was allowed before further procedures.

Mice were anesthetized with pentobarbital (200 mg/kg, i.p., Mebunat, Orion Pharma) and perfused transcardially with cold 1× PBS solution followed by 4% paraformaldehyde solution (PFA). Following decapitation the brains were collected to 15 mL falcon tubes (CLS430791, Corning) filled with the the same 4% PFA solution for overnight fixation. After, brains were transfered to 30% sucrose in 1× PBS until sinking (at least 48 h). The brains were then frozen on dry ice and stored at −80°C until sectioned. For the anterograde tracing, 80 μm coronal sections were collected throughout the whole brain, and for the retrograde tracing, 40 μm coronal sections from the injection site and the VTA were cut with a cryostat (CM3050S, Leica Biosystems).

To enhance the fluorescence signal of the anterotraced axons, anti-GFP immunostaining was performed. The sections were washed at room temperature in 1× PBS (5 min, three times), and blocked with 1% bovine serum albumin (Sigma-Aldrich) with 0.3% Triton X-100 (BDH Laboratory Supplies) in 1× PBS for 1 h at room temperature. The sections were then incubated with the primary antibody (chicken anti-GFP, 1:800 in blocking solution; catalog #ab13970, Abcam) overnight at 4°C, washed with 1× PBS (5 min, three times) and incubated with the secondary antibody (goat anti-chicken with Alexa Fluor 488, 1:800 in blocking solution; catalog #ab150169, Abcam) for 2 h at room temperature. Sections were then washed once more with 1× PBS (5 min, three times), mounted on microscope slides, and coverslips were applied with VECTASHIELD mounting medium (Vector Laboratories).

Imaging was performed with Zeiss Axio Imager Z2 using a 10× air objective in tiles mode. The successful targeting of the VTA was evaluated by two independent researchers using a mask drawn in Zen 3.1 software (Zeiss) based on previous immunohistochemical Th+ staining delineating the VTA. The Sst+ nature of the infected cells was also confirmed by the red inbuilt signal of tdTomato. Anterograde targets of the projecting cells were subjectively evaluated and classified as having no, low, medium, and strong GFP-expressing axonal arborizations. The conclusion about the consistency of the projection target was made according to hierarchical clustering results produced by *hclust* function from “stats” package implemented in R programming environment (https://www.rdocumentation.org/packages/stats/versions/3.6.2/). The script and the source data to reproduce the clustering can be found here at https://github.com/eLinanin/Anterograde_heatmap.git.

Retrogradely traced neurons within the VTA were manually counted and considered positive by having clear coexpression of the inbuilt tdTomato signal and the GFP signal caused by the viral infection.

### Electrophysiology

To define electrophysiological subtypes of the projecting VTA^Sst^ neurons, we combined backtracing with current-clamp recordings. Backtracing was performed as described above (see above, Tracing) on postnatal day (P)60–P90 Sst-IRES-Cre animals of both sexes. Electrophysiology was done after at least 1 month of recovery as described previously (Nagaeva et al., 2020) with some modifications. Shortly, mice were perfused with ice-cold constantly oxygenated NMDG-based cutting solution. Coronal VTA sections of 225 μm thickness were cut in the same solution using a vibratome (HM 650V, Thermo Fisher Scientific) and transferred to the constantly oxygenated 33°C HEPES-ACSF solution for 15 min recovery and then remained in the same solution at room temperature until the end of the experiment (∼3 h). For the injection site verification, sections were cut in the same way as the VTA sections and checked immediately with epifluorescence microscope BX51WI (Olympus).

Electrophysiological registration of the firing patterns was performed in the same conditions as previously described (Nagaeva et al., 2020) to allow data alignment. Backtraced VTA^Sst^ GFP-positive cells were identified with epifluorescence microscope BX51WI and a sCMOS (scientific complementary metal-oxide semiconductor) camera, Andor Zyla 5.5 (Oxford Instruments). Whole-cell current-clamp recordings were made using 3–5 MΩ borosilicate glass electrodes filled with 1–2 μl of intracellular solution (IS) containing the following (in mm): 130 K-gluconate, 6 NaCl, 10 HEPES, 0.5 EGTA, 4 Na2-ATP, 0.35 Na-GTP, and 8 Na2-phosphocreatine. pH adjusted to 7.2 with KOH, osmolarity ∼285 mOsm. Liquid junction potential (+12 mV) was not corrected during recordings.

All electrophysiological experiments were made with a MultiClamp 700B amplifier (Molecular Devices) filtered at 2 kHz, and recorded with a 10 kHz sampling rate using pClamp 10 software (Molecular Devices). After achieving whole-cell configuration in voltage-clamp mode (−70 mV), cell capacitance was determined by the Membrane Test feature of the Clampfit software, and the amplifier was then switched to current-clamp mode. Depolarized cells with a resting membrane potential higher than −50 mV were excluded. For measuring passive and active membrane properties, neurons were injected with 800 ms current steps with 10 pA increments, resulting in the membrane voltage fluctuation from −120 mV to the saturated level of firing. In addition, a shorter protocol from 0 pA with 1 pA increment was applied for better identification of the action potential threshold and shape. All recordings were performed with intact GABAergic and glutamatergic transmission (i.e., no pharmacological agents were added to the ACSF).

### Caspase experiments

For the caspase manipulations, Sst-tdTomato male and female mice P60–P75 (on the injection day) were used. All the injection procedures were similar to those described (see above, Tracing). Slightly modified coordinates for the VTA were used, aiming at the anterolateral Sst+ subpopulation (Nagaeva et al., 2020) and based on the results of the retrograde tracing to hit the majority of the projecting VTA^Sst^ neurons (mm relative to bregma), AP −3.1, ML ±0.7, DV −4.6. Bilateral injections (600 nl) of a 1:1 mixture of AAV1/2-DIO-taCasp3-TEV (3 × 10^12^ genome copies/ml, a gift from Prof. William Wisden) and AAV2/8-CAG-Flex-Myr-eGFP (4 × 10^12^ genome copies/ml; Neurophotonics Center, CERVO Brain Research Centre) viral constructs, or 1:1 dH_2_0 dilution of AAV2/8-CAG-Flex-Myr-eGFP for the controls, were made with a flow rate of 0.1 μl/min. Behavioral experiments started 4 weeks after surgeries to ensure successful viral expression.

Once all behavioral tests were conducted, mice were perfused and brains collected (see above, Tracing) to confirm the successful deletion of VTA^Sst^ neurons. Forty-μm-thick coronal sections containing the VTA area were made and imaged with the epifluorescence microscope. Mice in which the viral injection did not reach the VTA or was too lateral or too medial were excluded from the analysis (four mice from cohort 1, two from cohort 2, and five mice from the IntelliCage cohort). The subjective exclusion was performed by two independent researchers as described above. In addition, numbers of the eGFP-expressing cells in the VTA of five control and five caspase brains from both sexes randomly selected were counted and analyzed.

### Behavioral experiments

#### General description

Seventeen (9 male, 8 female) VTA^Sst−^ and 21 (10 male, 11 female) control mice represented by two cohorts underwent the same battery of behavioral tests to study reward- and anxiety-related behaviors. The timeline of the experiments is shown in Extended Data Figure 5-1*d*, and it took into consideration possible behavioral side effects of the performed tests (e.g., the morphine sensitization test was terminal for both cohorts). Experiments were performed in the morning between 7:00 A.M. and 1:00 P.M. Mice were always habituated to the testing room 45–60 min before the experiment. Arenas were cleaned thoroughly with water between animals. Researchers conducting the experiments were blinded to the treatment group of the animals. Mice were initially group housed but were then single housed (including 3 d of habituation) for conducting the novelty-suppressed feeding (NSF), sucrose preference, running wheel activity, and morphine sensitization tests (Extended Data Fig. 5-1*d*, timeline). To reduce stress from the injection, mice underwent a 5 d habituation routine before morphine injections as described previously (Elsilä et al., 2022).

#### Novelty-induced locomotor activity and morphine sensitization

The experiment was performed as described previously (Vashchinkina et al., 2012). Shortly, mice were released one by one in the novel open arena (36 × 19 × 20 cm). Distance moved was recorded during 60 min with EthoVision XT 10 tracking equipment (Noldus). Illumination in the room was ∼50 lux.

For morphine sensitization, each mouse was habituated to the arena for 60 min after which it received a morphine injection (20 mg/kg, i.p.) and was immediately placed back in the arena. Mice remained in the arena for 3 h, and locomotor behavior was monitored using EthoVision tracking software (induction day). After the testing, the mice were put back in their home cage. Mice were challenged with a morphine injection (20 mg/kg, i.p.) 7 d later, and the experiment was repeated in the same context (challenge day). Sensitization to the morphine-enhanced locomotor activity was calculated as the difference of distance moved on the challenge day minus distance moved on the induction day. Two male caspase-treated mice were excluded from the analysis because of the problems with the first (induction) morphine injection. Exclusion did not change the statistical outcome.

#### Elevated plus maze (EPM)

The elevated plus maze was made out of gray plastic and consisted of a central platform (5 × 5 cm), from which two open arms and two enclosed arms (5 × 40 × 20 cm) extended at an elevation of 50 cm from the floor (Lister, 1987). The light intensity of the closed arms was 10 lux and open arm 200 lux. The mouse was placed on the central platform facing a closed arm and allowed free exploration of the maze for 5 min. Distance moved and time spent in different arms was recorded with EthoVision. Time spent in the open arm was calculated as the percentage of the total time spent in all arms.

#### Light/dark box (LDB)

The test was performed in an open-field arena (43.2 × 43.2 × 30.5 cm; catalog #ENV-515, Med Associates) equipped with infrared light transmitters and sensors detecting horizontal and vertical activity. The dark insert (nontransparent for visible light) was used to divide the arena into two equally sized compartments. An open door (width of 5.5 cm and height of 7 cm) in the wall of the insert allowed the animal to freely move between compartments. Illumination in the light compartment from bright ceiling lights was ∼200 lux. The animal was released in the door, head facing the dark compartment, and allowed to explore the arena for 5 min. Distance moved and time spent in different compartments were recorded by the system. Time spent in the light compartment was calculated as the percentage of the total time spent in both compartments.

#### Forced-swim test (FST)

The mouse was placed for 6 min in a glass beaker (diameter 15 cm, height 25 cm) filled with 3 L of water at 23 ± 1°C (Procaccini et al., 2011). Three visually isolated mice were recorded simultaneously using a digital video camera. Latency to the first immobility and time of total immobility (i.e., passive floating, when an animal was motionless and only doing a slight movement with a tail or one hindlimb, in contrast to struggling, climbing or swimming with all four paws) were measured manually in 2 min intervals by a blinded researcher.

#### Novelty-suppressed feeding (NSF)

Mice were single-housed and food-restricted for 14 h before testing to motivate food-seeking behavior. Water was given *ad libitum*. Mice were tested in an open arena (50 × 50 × 28 cm) at an illumination of 200 lux. A lid from a 50 ml falcon tube placed in the middle of the arena contained a small amount of moist food. The mouse was released next to the wall, and its behavior was recorded using EthoVision. Latency until the mouse started eating (i.e., eating for >5 s) was monitored manually by a researcher, after which the trial was terminated (maximum cutoff point 10 min). The mouse was returned to its home cage, where it was again presented with a small amount of moist food. Latency until first in-cage eating was monitored as previously. This was done to account for possible differences in hunger between animals.

#### Sucrose preference test

A two-bottle choice sucrose preference test was conducted for 7 d (Lainiola et al., 2019). Mice were single housed in larger IVC cages allowing the use of two drinking bottles. Drinking bottles were weighted daily at 10:00 A.M. to monitor consumption. The position of the water and sucrose bottles was changed every day to avoid the development of side preference. Mouse body weights were measured before and after the start of the experiment. Sucrose concentrations were based on an earlier study analyzing sucrose preference in C57 mice (Sclafani, 2006) and were increased as follows: 0.1% (2 d), 0.5% (2 d), and 1% sucrose (3 d). The average was taken for each sucrose concentration. Sucrose preference was calculated as a percentage of sucrose consumption of the total fluid intake.

#### Circadian rhythm of running wheel activity

To measure voluntary wheel running activity, free-running wheels (Med Associates) were placed in the IVC cage of a single-housed mouse. The rotation of the wheel by the mouse was transmitted as a digital signal wirelessly to a hub and recorded on Wheel Manager software. Data were exported every morning, and running wheels were checked for proper functioning. Voluntary running wheel activity was followed for 3 d; the first day was considered as habituation followed by 2 d of basal activity. Ten mice, which did not use the running wheel and ran <100 rotations over a course of 3 d, were excluded from the analysis.

#### IntelliCage (IC)

A separate IntelliCage cohort of originally 16 (8 male, 8 female) VTA^Sst−^ and 15 (7 male, 8 female) control mice was subcutaneously injected with RFID transponders (Planet ID) for individual identification. The IntelliCage by NewBehavior (TSE Systems) is an apparatus designed to fit inside a large cage (610 × 435 × 215 mm; Tecniplast 2000P). The apparatus itself provides four recording chambers that fit into the corners of the housing cage. Access into the chambers was provided via a tubular antenna (50 mm outer and 30 mm inner diameter) reading the transponder codes. The chamber contains two openings of 13 mm in diameter (one on the left, one on the right), which gave access to drinking bottles. These openings are crossed by photograph beams recording nose pokes of the mice, and the holes can be closed by motorized doors. Four triangular red shelters (Tecniplast) were placed in the middle of the IntelliCage and used as sleeping quarters and as a stand to reach the food. The floor was covered with a thick (2–3 cm) layer of bedding. The IntelliCage was controlled by a computer with dedicated software (IntelliCagePlus), executing preprogrammed experimental schedules and registering the number and duration of visits to the corner chambers, nose pokes to the door openings, and lickings as behavioral measures for each mouse. To randomize treatment groups and allow noncompetitive access to the corners, mice were housed in four IntelliCages in balanced groups of the same sex (e.g., four control plus four caspase). The mice were group housed in these groups from weaning (cage type Tecniplast Green Line GR900), at least 10 weeks before the start of IntelliCage experiments. All tests in the IntelliCage system were conducted in the order they are listed below on the consecutive days without taking mice out of the cages except on 2 cleaning days.

##### Free adaptation

At the beginning of the test, the mice were released in the IntelliCage during the light phase at 9:00 A.M. with all doors open allowing unlimited access to the water bottles (free adaptation). Animals were allowed to explore the new environment for 3 consecutive days. The exploratory, locomotor, and circadian activities were measured as a number of corner visits or as nose pokes to the water bottles per hour for each day separately (day 1, adaptation phase; days 2 and 3, basic activity). Similarly, drinking behavior was measured as the number of licks/h.

##### Adaptation to nose poke

On the fourth day, all doors were closed at the beginning of the experiment, and mice were required to poke into closed gates to reach drinking tubes. Only the first nose poke of the visit opened the door for 5 s (predefined time). Animals had to start a new visit to get access to water again. This rule was the same in all experiments requiring nose poking.

##### Saccharine preference

In this task, all four corners operated the same way, 24 h per day; doors opened spontaneously for a 7 s drinking period on the entry to a corner. Each corner contained a bottle of saccharine on one side and a bottle of water on the other. High- and low-saccharine concentrations were chosen based on previous research (Pijlman et al., 2003; Sclafani et al., 2010). Every day, saccharine and water sides were alternated to exclude side preference. During the first 3 d, mice were suggested to choose between two low saccharine concentrations 0.01% (S1) and 0.03% (S2) assigned to two opposite corners each. During the next 3 d, the lowest 0.01% (S1) concentration was exchanged for the highest 0.3% (S3) one, and the order of the corners was changed as well. For a better understanding of the schedule, a simplified scheme with the corners and side assignments is depicted in Extended Data Figure 8-1*a*. The preference score was calculated as a percentage of the number of licks to a certain liquid (two saccharine liquids of different concentrations and two water liquids in corresponding saccharine corners) from the total number of licks during the last 2 d of each session.

##### Delay discounting

In this experiment, all four corners were accessible to all animals and contained 0.3% saccharine liquid (S3) on one side of the corner and water on the other side. The order of the bottles was as follows: S3, 1, 3, 6, 8; water, 2, 4, 5, 7. On day 0 both doors to the water and saccharine opened simultaneously for 7 s on entry to the corner. On day 1, saccharine door opened with a 0.5 s delay, and the water bottle door opened immediately. The next day, the delay before the opening of the saccharine door increased to 1 s and then for 1 s every next 24 h. After 4 d, this resulted in a delay of 5 s. A saccharine preference score was calculated as a percentage of the lick number to saccharine bottles from a total lick number (saccharine plus water).

##### Saccharine extinction and avoidance

In these two tasks, the setup was very similar in general and differed only in the third phase. Water was always available on the entry in two corners for all animals. Saccharine 0.3% (S3) was available with a rule in one corner for four specific mice (two caspase plus two controls) and in another corner for the remaining four mice to avoid competition. The rule was as follows: To get the saccharine mice had to nose poke in one of the side doors, which triggered the LED light above this door for 1.5 s, and then the door opened after an extra 0.5 s delay for 5 s. Mice had to repeat the sequence to get additional access to the saccharine. In Phase 1 (33 h), mice were learning the rule; in Phase 2 (48 h) mice were adapted to the rule, and the basic activity was measured; in Phase 3 (38 h) saccharine bottles were emptied for the Extinction experiment. In the Avoidance experiment mice went through the same sequence of events, but in Phase 1 saccharine bottles were back, and the order of the corners was changed, so they had to learn new rules (relearning). In Phase 3 (Avoidance), mice received a 0.2 bar air puff in the saccharine door with a 25% probability. The activity was measured during the whole experiment as a number of nose pokes/h. Similarly, drinking behavior was measured as licks/h.

#### Fear conditioning

For these experiments, the same IntelliCage cohort of mice was used, and this was a terminal experiment for this cohort of mice. Animals were single housed after the IntelliCage experiments and given 2 weeks of adaptation before the fear conditioning (FC). The FC protocol was based on previously published studies (Mennesson et al., 2020) with few modifications and consisted of three phases, acquisition, context test, and cue test. Briefly, mice were placed one by one in the test chamber (Video Fear Conditioning, Med Associates) for fear acquisition and conditioning. After 120 s of free exploration, a 30 s 5 kHz 90 dB cue tone sounded from the wall-mounted speakers, coterminated with a 2 s scrambled 0.6 mA shock through the grid floor. Cue-shock pairs were repeated twice again with 90 and 60 s intertrial intervals; after that the session was finished with 120 s of free exploration. Chamber light, near-infrared light, and a fan were on during all phases. After each mouse the chamber was cleaned with water.

Nine days later, the mice were tested in the same chambers for the context-induced retrieval of the fear memory. For that, the mouse was placed in the same testing chamber with identical conditions as before, except for no cue tones or shocks, for 300 s of free exploration.

Five hours after the context test, cue-induced retrieval of the fear memory was assessed in the conditioning chamber with the floor and wall material and the shape of the chamber changed to exclude the context component. After 120 s of free exploration, the mouse was introduced to 20 30 s cue tones (identical to those used during conditioning) separated by 5 s intertone intervals. After each mouse, the chamber was cleaned with 70% ethanol.

Freezing time and the number of freezing episodes were automatically analyzed by Video Freeze Software (Med Associates) separately for each component of the test phases. Two female mice had missing data for the acquisition phase, but they still received the footshock and were included in the context- and cue-induced memory retrieval data analysis.

### Drugs

Morphine hydrochloride (University Pharmacy) was dissolved in physiological saline (0.9% NaCl) on the day of treatment. Morphine was injected in a volume of 10 ml/kg body weight.

### Statistics

For behavioral experiments, statistical analysis was done using IBM SPSS Statistics version 28.0.0.0. whereas graphs were drawn with GraphPad Prism version 8.1.10. The data were tested for normality and homogeneity of variance using the Kolmogorov–Smirnov and Levene’s tests, respectively. When assumptions were violated, the square root transformation was applied (elevated plus maze and running wheel experiments). Statistical analyses of the data were done using univariate and repeated-measures two-way ANOVAs unless stated otherwise. In case of significant main effect or interaction, *post hoc* tests were performed using a multiple comparisons test with Bonferroni correction. The level of significance was set at 0.05. All data are shown as mean ± SEM.

## Results

### VTA^Sst^ neurons innervate several forebrain regions

**Figure 1.**
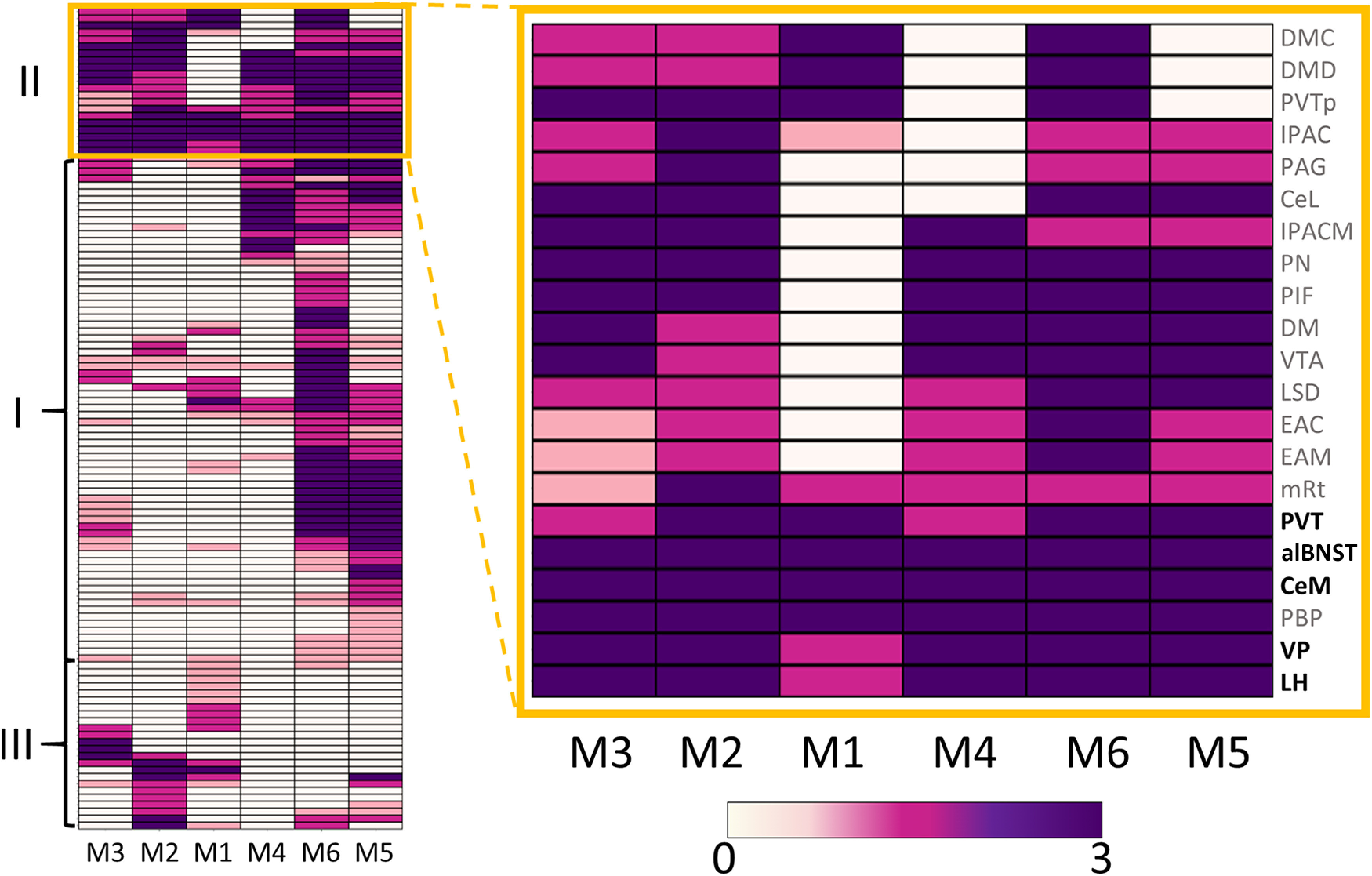
Heat map of the VTA^Sst^ neuron projections. Left, The heat map and the clustering results of all regions receiving GFP+ projections in six VTA-injected Sst-Cre animals, includes three clusters (I–III) defined by the algorithm. Right, The yellow frame contains the enlarged image of the cluster of the brain regions with the densest innervation (cluster II) in all six animals. The *y*-axis depicts the names of the regions, and the *x*-axis shows the mouse number (M1, mouse 1, etc.) The names of the areas that had consistently the strongest fluorescent signal in all six animals as defined by the heat map are marked in bold in the far right column. Colors indicate the density of the fluorescent axons in the projection site from 0 (no projections) to 3 (highest density). Left, The script and the source data to reproduce the clustering and see the names of the rest brain regions can be found at https://github.com/eLinanin/Anterograde_heatmap.git. All source data can be found in Extended Data 1. CeL, Central amygdala, lateral part; DM, dorsomedial hypothalamic nucleus; DMC, DM compact part; DMD, DM dorsal part; EAC, extended amygdala central part; EAM, extended amygdala medial part; IPAC, interstitial nucleus of the posterior limb of the anterior commissure; IPACM, IPAC medial part; LSD, lateral septal nucleus dorsal part; mRT, mesencephalic reticular formation; PAG, periaqueductal gray; PBP, parabrachial pigmented nucleus of the VTA; PIF, parainterfascicular nucleus of the VTA; PN, paranigral nucleus of VTA; PVTp, PVT, posterior part; VP(mfb) VP medial forebrain bundle.

**Figure 2.**
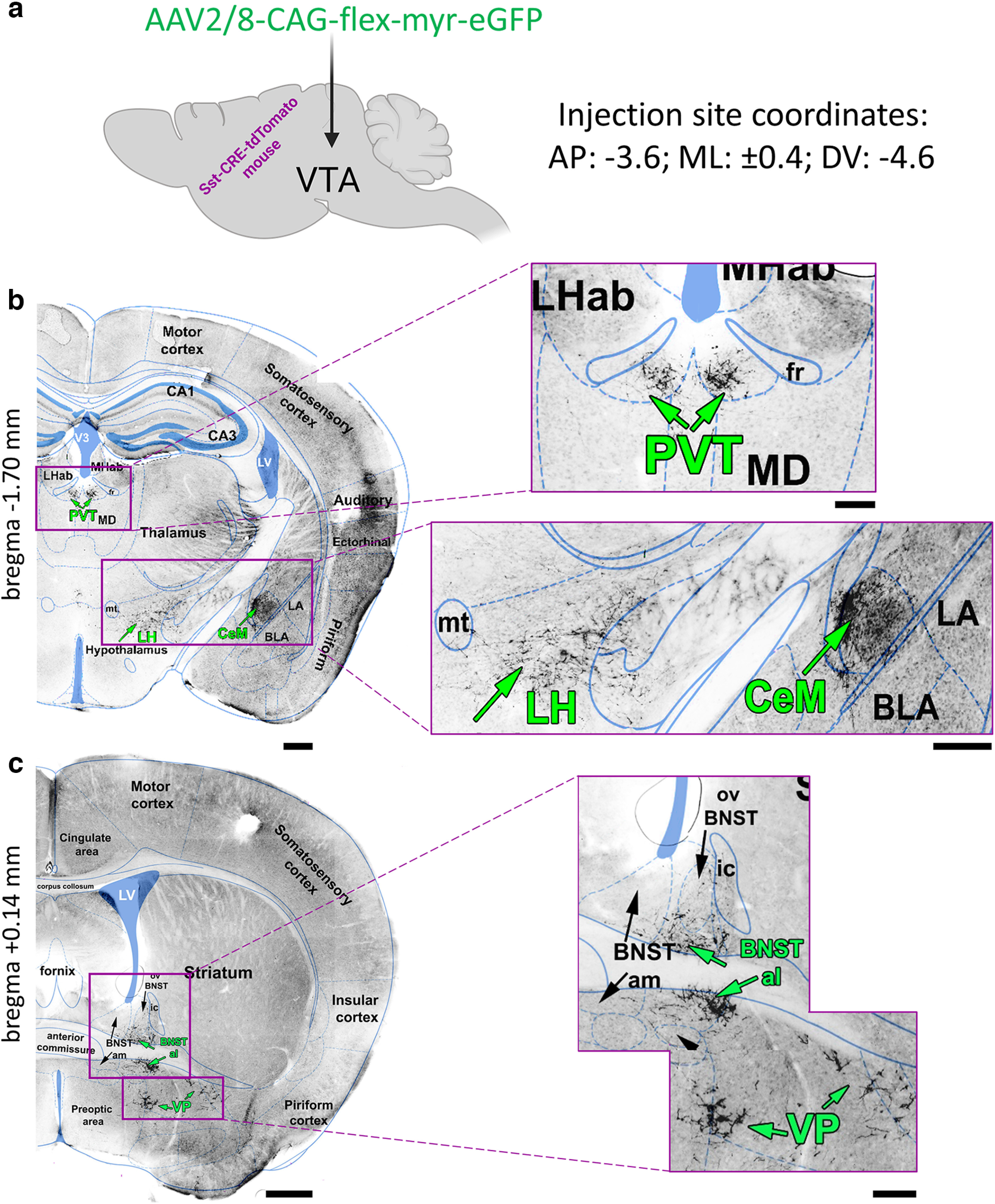
Anterograde tracing of the VTA^Sst^ neurons. ***a***, Sst-tdTomato mice received a unilateral intra-VTA injection of a Cre-dependent AAV tracer. The scheme shows the name of the viral tracer and injection coordinates. ***b***, Examples of the VTA^Sst^ projections found in the PVT, LH, and CeM at the bregma level −1.70 mm. ***c***, Examples of the VTA^Sst^ projections found in the alBNST and VP at the bregma level +0.14 mm. Images in ***b*** and ***c*** are the black-and-white variants of the fluorescent GFP+ images of the coronal mouse brain sections (Fig. 1, mouse 1). Scale bars: ***b***, ***c***, left, black lines: 500 μm; right: 200 μm. amBNST, anteromedial part of BNST; BLA, basolateral amygdala; CA1 and CA3, hippocampal areas CA1 and CA3; fr, fasciculus retroflexus; ic, internal capsule; LA, lateral amygdala; LHab and MHab, lateral and medial habenula; LV, lateral ventricle; MD, mediadorsal thalamic nucleus; mt, mammillothalamic tract; ovBNST, oval BNST; V3, ventricle 3.

To find out whether Sst+ neurons project outside the VTA, we injected a Cre-dependent anterograde tracer unilaterally into the VTA of Sst-Cre mice and sectioned the whole brain 3 weeks later to locate the GFP signal of the tracer. We found VTA^Sst^ projections in 118 brain regions among six studied animals. Then we defined the regions that consistently had the densest axonal arborizations in all the brains studied using hierarchical clustering and depicted the results as a heat map (Fig. 1). VTA^Sst^ neurons were found to have five consistent projection targets, the VP, LH, CeM, alBNST, and PVT (Figs. 1, 2).

10.1523/ENEURO.0149-23.2023.ext1 Extended Data 1 Source data tables. Download Extended Data 1, XLS file.

**Figure 3.**
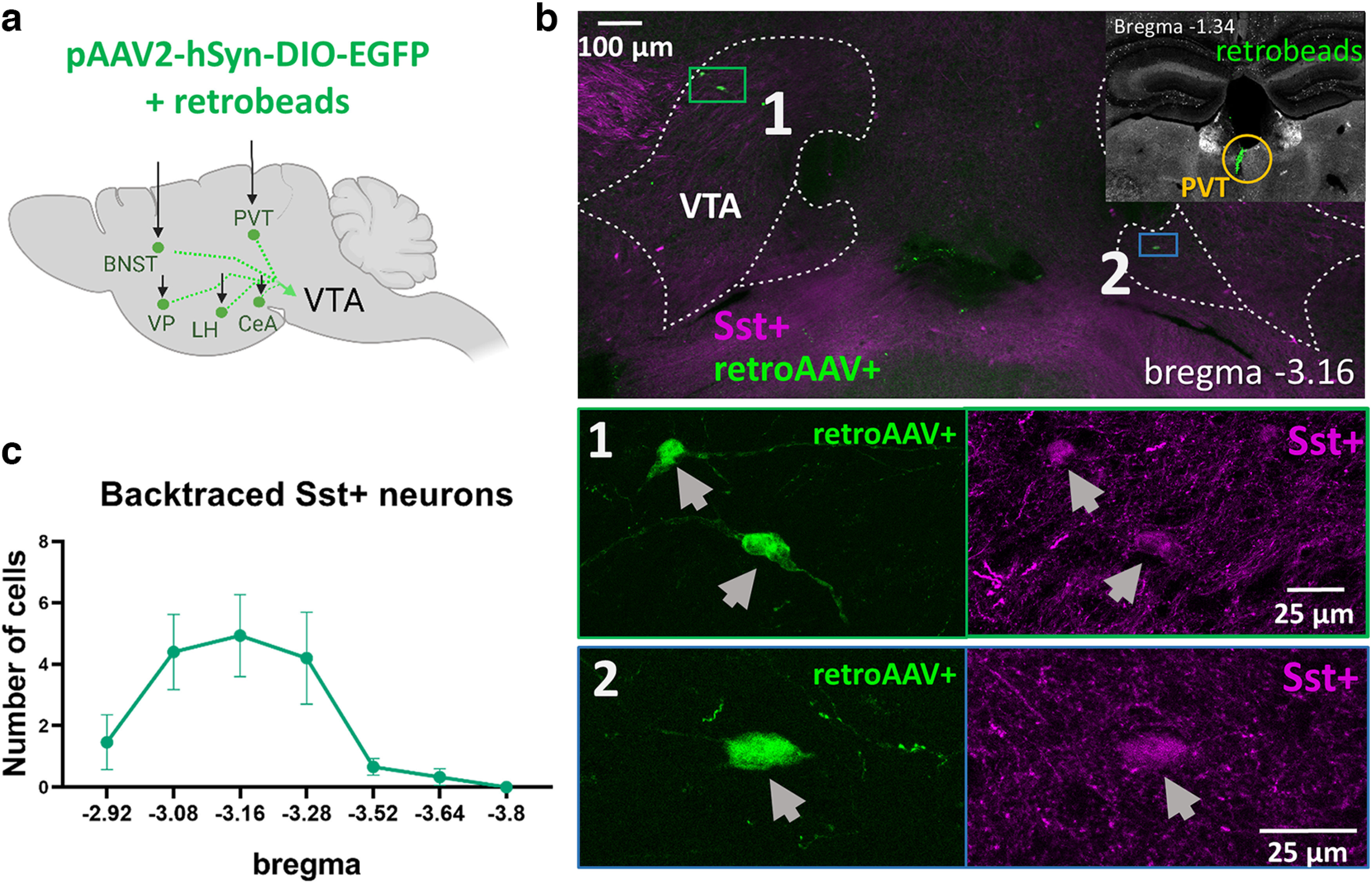
Retrograde tracing of the VTA-projecting Sst+ neurons. ***a***, Sst-tdTomato mice each received a unilateral injection of the mixture of the Cre-dependent retro-AAV virus and RetroBeads in one of the five found VTA^Sst^ projection targets (Fig. 2). ***b***, Examples of the backtraced neurons in the VTA at the bregma level −3.16 mm. Top right, RetroBeads (green dots) in the injection site within the PVT (yellow circle). The green rectangle shows ipsilaterally traced neurons (1), and the blue rectangle shows a contralaterally traced neuron (2). Bottom, Magnified images (1 and 2) of the green and blue rectangles. Retrograde tracings of LH, CeM, alBNST, and VP are shown in Extended Data Figures 3-1, 3-2, 3-3 and 3-4. ***c***, The graph shows the distribution of the backtraced VTA^Sst^ neurons at different bregma levels. The number of the backtraced Sst+ neurons from five different targets (LH, CeM, PVT, alBNST, and VP) were combined and are shown as average ± SEM (*n* = 15 mice).

Some of the projection targets, such as the LH and VP, are along the way of the medial forebrain bundle, the massive neuronal tract connecting the midbrain with the forebrain. To confirm that VTA^Sst^ axons innervate the targets mentioned and not only pass through the area, we injected a Cre-dependent retrograde tracer unilaterally into each of these targets (Fig. 3*a*). Indeed, injections into the CeM, LH, alBNST, and VP produced GFP expression in the cell bodies of VTA^Sst^ neurons located ipsilaterally to the injection site (Extended Data Figs. 3-1, 3-2, 3-3, 3-4). However, unilateral injection of the retrotracer into the PVT produced GFP expression bilaterally in the VTA (Fig. 3*b*). Similarly, we detected traced axons in the left and right parts of the posterior PVT after unilateral VTA injection of the anterograde tracer (Fig. 2*b*). This might suggest that either individual VTA^Sst^ neurons send collaterals to the right and left parts of the PVT, or different Sst+ neurons from the same VTA side innervated the PVT bilaterally. Another explanation may lie in the nonbilateral anatomy of the PVT, which is a member of the midline thalamic nuclei family (Kirouac, 2015).

10.1523/ENEURO.0149-23.2023.f3-1 Figure 3-1 Backtracing from the medial part of the central amygdala. Examples of the backtraced Sst+ neurons in the VTA at the bregma level −3.08 mm in Sst-tdTomato (magenta) mouse. Right, Image shows RetroBeads in the injection site (CeM). The yellow circle shows the actual unilateral injection spot. Top left, Green rectangle shows ipsilaterally traced neurons. Bottom, Magnified images of the green rectangle. BLA, Basolateral amygdala; CeL, lateral part of the central amygdala; PBP, parabrachial pigmented nucleus of the VTA. Download Figure 3-1, TIF file.

10.1523/ENEURO.0149-23.2023.f3-2 Figure 3-2 Backtracing from the anterolateral part of the bed nucleus of stria terminalis. Examples of the backtraced Sst+ neurons in the VTA at the bregma level −3.08 mm in Sst-tdTomato (magenta) mouse. Right, Image shows RetroBeads in the injection site (alBNST). The yellow circle shows the actual injection spot. Top left, Green rectangle shows ipsilaterally traced neurons. Bottom, Magnified images inside green rectangle split by fluorescent channels. ac, Anterior commissure; cc, corpus callosum; CPu, caudatus-putamen (striatum); ml, medial lemniscus; PBP, parabrachial pigmented nucleus of the VTA. Download Figure 3-2, TIF file.

10.1523/ENEURO.0149-23.2023.f3-3 Figure 3-3 Backtracing from the lateral hypothalamus. Examples of the backtraced Sst+ neurons in the VTA at the bregma level –3.28 mm in Sst-tdTomato (magenta) mouse. Top right, RetroBeads at the injection site (LH). The yellow circle shows the actual unilateral injection spot. Top left, Green rectangle shows an ipsilaterally traced neuron. Bottom, Magnified images inside the green rectangle split by fluorescent channels. ic, Internal capsule; ml, medial lemniscus; PBP, parabrachial pigmented nucleus of the VTA; PN, paranigral nucleus of VTA. Download Figure 3-3, TIF file.

**Figure 4.**
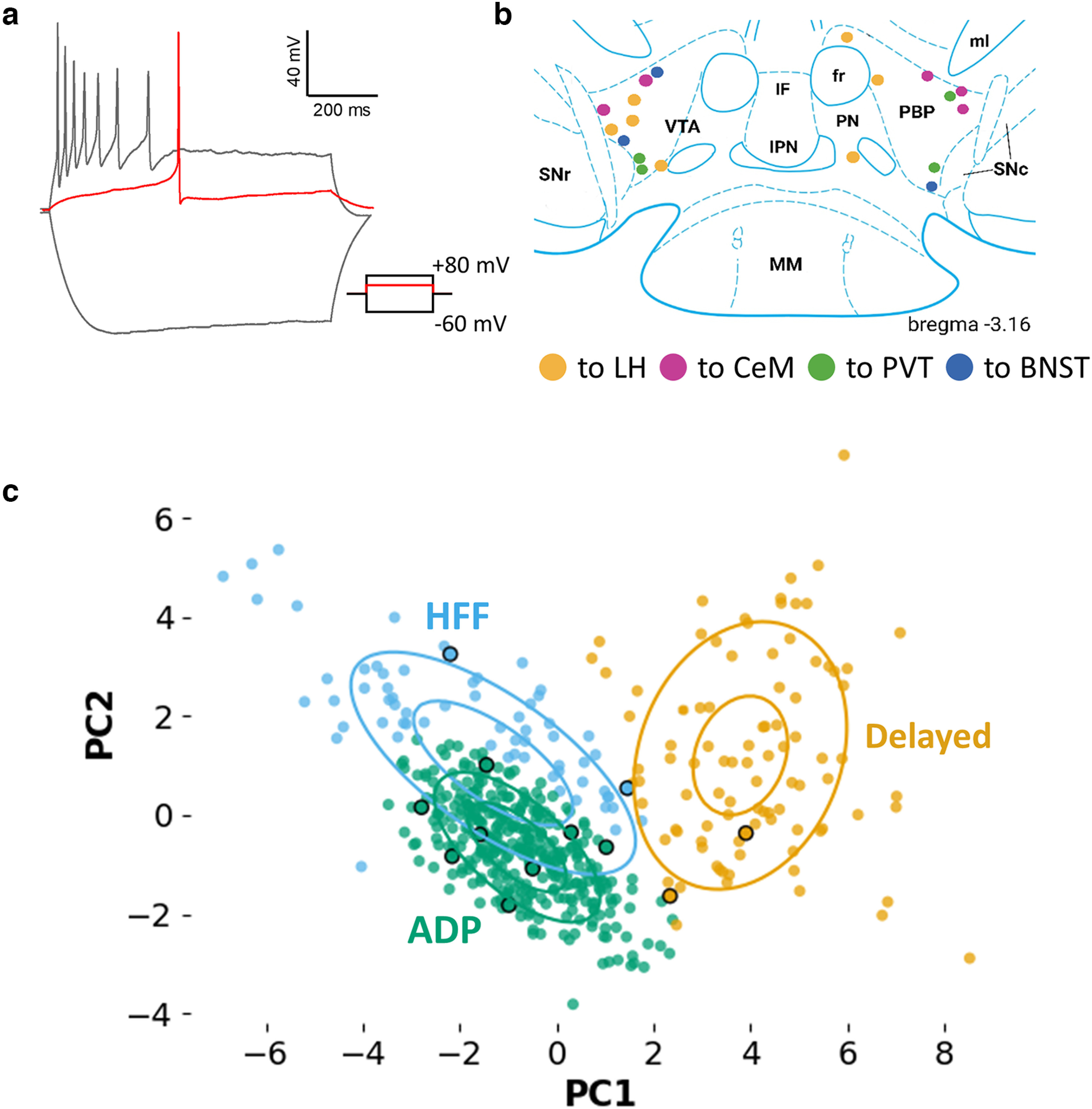
Electrophysiological profile and location of VTA^Sst^ neurons projecting to the forebrain. ***a***, A representative trace of the response of a VTA^Sst^-projecting neuron to injected 800 ms current steps of −60, +8 (red line), and +80 mV. Adapting firing pattern at −80 mV and no delay before the firing resemble features of the ADP Sst+ subtype (Nagaeva et al., 2020). ***b***, Locations of the recorded VTA^Sst^ neurons at the bregma level −3.16 mm. Their projection sites are color coded. Locations at bregma levels −3.08 and −3.28 are shown in Extended Data Figure 4-1. ***c***, Most of the recorded backtraced VTA^Sst^ neurons (circled in black) were assigned to the ADP cluster by unsupervised clustering procedure with a previously published dataset of the VTA^Sst^ neurons as the reference (method and clusters described in Nagaeva et al., 2020; Fig. 3). IF, interfascicular nucleus; IPN, interpeduncular nuceus; ml, medial lemniscus; MM, medial part of medial mammillary nucleus; SNr, substantia nigra, reticular part; SNc, substantia nigra, compact part.

10.1523/ENEURO.0149-23.2023.f3-4 Figure 3-4 Backtracing from the ventral pallidum. Examples of the backtraced Sst+ neurons in the VTA at the bregma level −3.08 mm in Sst-Cre mouse. Right, The image shows RetroBeads in the injection site (VP). The yellow circle shows the actual unilateral injection spot. Top left, Green rectangle shows ipsilaterally traced neurons. Bottom, The magnified image of the green rectangle. ac, Anterior commissure; cc, corpus callosum; CPu, caudatus-putamen (striatum); PBP, parabrachial pigmented nucleus of the VTA. Download Figure 3-4, TIF file.

10.1523/ENEURO.0149-23.2023.f4-1 Figure 4-1 Location of the electrophysiologically recorded VTA^Sst^ neurons projecting to forebrain regions and their electrophysiological subtypes. ***a***, None of the backtraced neurons in electrophysiological experiments were found more posterior than the bregma level −3.28 mm, and most of them were located in the lateral nuclei of the VTA. Their projection sites are color coded. Download Figure 4-1, TIF file.

### The majority of VTA^Sst^-projecting neurons belong to the electrophysiological afterdepolarizing subtype

Retrograde tracing experiments showed location specificity of Sst+-projecting neurons in the anterolateral VTA. Most (225/240 analyzed) of the backtraced neurons were found in the anterolateral part of the VTA at the bregma levels between −2.9 and −3.3 mm, with more than half the cells (140 of 240) found at the bregma levels −3.08 and −3.16 mm (Figs. 3*c*, 4*b*; Extended Data 1). We previously demonstrated that different electrophysiological subtypes of VTA^Sst^ neurons have distinct locations, assigning anterolateral VTA neurons to either afterdepolarizing (ADP) or high-frequency firing (HFF) subtypes (Nagaeva et al., 2020). Therefore, we took an effort to define the electrophysiological subtype of the projecting neurons. To do that, we repeated the same procedure that was used for the backtracing experiments and performed patch-clamp recordings on GFP+ VTA neurons.

For the classification of electrophysiological subtypes of projecting Sst+ cells, we applied automatic firing pattern analysis (Nagaeva et al., 2021) and clustering algorithm (Nagaeva et al., 2020). We used our previously published electrophysiological dataset containing the firing patterns of 389 VTA^Sst^ neurons as the reference dataset in the clustering procedure (Nagaeva et al., 2020 contains full description of method and reference dataset). The majority (67%) of the backtraced neurons were assigned to the ADP cluster (Fig. 4*c*). Indeed, these neurons showed adapting firing rates at the saturated level of excitation, sag depolarization, and small afterdepolarization in the first rheobase action potential (Fig. 4*a*), mimicking the ADP Sst+ subtype. Anterolateral location of the recorded neurons (Fig. 4*b*; Extended Data Fig. 4-1) also supported their affiliation with the ADP subtype.

### Behavioral consequences of the deletion of VTA^Sst^ neurons

We used a loss of function approach to elucidate the behavioral impact of VTA^Sst^ neurons and selectively deleted them by injecting a Cre-dependent caspase-expressing virus bilaterally into the VTA area of adult Sst-Cre-tdTomato mice. Once expressed, this virus started the programmed cell death and eliminated Sst+ neurons exclusively in the injected region (Yang et al., 2013; Yu et al., 2019), resulting in VTA^Sst−^ mice. To visualize successful deletion, a GFP-expressing Cre-dependent virus was injected either together with the caspase virus or alone into the control group (Extended Data Fig. 5-1). We aimed to delete neurons preferably in the anterolateral VTA, where most of the projecting Sst+ neurons were found (Fig. 3*c*).

For the proper planning of the behavioral experiments, we considered both the previously established function of ADP VTA^Sst^ neurons as interneurons (Nagaeva et al., 2020) and the physiological function of their projection targets. As, on the one hand, VTA^Sst^ ADP neurons can inhibit locally neighboring DA neurons, we used several reward and motivation-related tasks to find out how the impairment of the local inhibitory circuitry affects these processes (Corre et al., 2018; Van Zessen et al., 2012). On the other hand, because they project to forebrain targets such as the PVT, CeM, LH, VP, and BNST implicated in the processing of the aversive stimuli and defensive behaviors (Gao et al., 2020; Gomes-de-Souza et al., 2021; Keifer et al., 2015; Lebow and Chen, 2016), we conducted stress-, anxiety- and fear-related behavioral tests. It was also important to find out whether there were any sex differences in the affected behaviors as several VTA^Sst^ projection targets, such as the PVT, BNST and LH, are known to be sexually dimorphic structures (Kim et al., 2017; López-Ferreras et al., 2017; Uchida et al., 2019).

### Increase in home-cage activity of VTA^Sst−^ female mice

**Figure 5.**
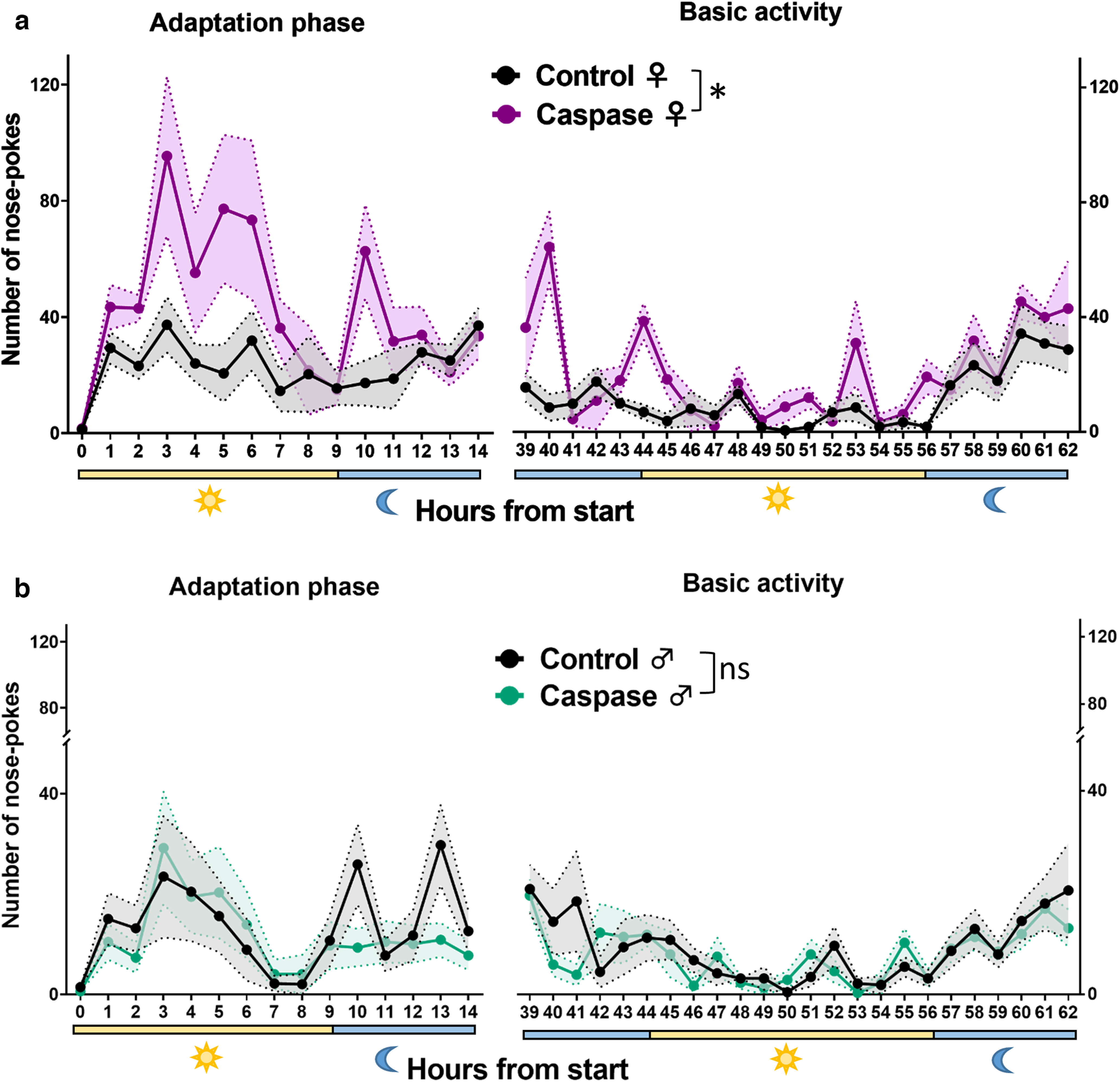
VTA^Sst^-caspase female mice demonstrated an increased number of nose pokes in the IntelliCage system. To study the behavioral functions of VTA^Sst^ neurons, a Cre-dependent caspase-expressing virus was injected bilaterally into the VTA resulting in VTA^Sst−^ mice (Extended Data Fig. 5-1). ***a***, The *y*-axis indicates the number of nose pokes into the water-containing doors per hour; the *x*-axis shows the time after the beginning of the test. Bottom, Yellow and blue bars show light and dark phases, respectively. VTA^Sst^-caspase females nose poked more often in the IntelliCage environment than the control females, suggesting higher activity during the adaptation (sex × treatment, *F*_(1,22)_ = 7.085, *p* = 0.014; female, *post hoc*, *p* = 0.004) and after habituation to the new environment (sex × treatment *F*_(1,22)_ = 8.043, *p* = 0.010; female, *post hoc*, *p* = 0.002). Females were overall more active than males (sex, *F*_(1,22)_ = 27.329, *p* < 0.001). ***b***, There was no difference between the male treatment groups. Classical anxiety tests and circadian activity in the free-running wheel test in single housing did not reveal any differences (Extended Data Figs. 5-2, 5-3). Statistical analyses of all behavioral tests are presented in Extended Data Table 5-1. Data are shown as mean ± SEM,* *p* < 0.05 for the significance of the difference in overall activities (*post hoc* test). ns - no significant difference, *p* > 0.05.

Table 1 contains information on the behavioral experiments, the numbers of animals tested in the control and VTA^Sst−^ groups, and whether we found statistically significant differences in behavior. Of the 18 tests performed, 5 tests revealed differences between the VTA^Sst−^ and control groups. Interestingly, some of the differences were sex specific. The VTA^Sst−^ females were more active in nose poking to the water-containing doors in the corners during the first 14 h in the IntelliCage environment than the control females (Fig. 5*a*). Their activity remained upregulated after 39–62 h of adaptation to the IntelliCage, suggesting that the difference was not because of exploration of the novel environment. There were no changes in this activity between the control and VTA^Sst−^ male mice (Fig. 5*b*). It is important to note that the number of licks to water bottle tips behind the doors was similar in all mouse groups, indicating no increase in water consumption in the VTA^Sst−^ females or in the females overall compared with males (Extended Data Table 5-1). Similarly, we did not see any differences in the open-field and anxiety tests between the control and VTA^Sst−^ mice (Extended Data Fig. 5-2), suggesting that deletion of VTA^Sst^ neurons influenced exclusively the home-cage activity in females and not the explorative activity or the level of anxiety.

**Table 1:** Results of behavioral tests for VTA^S^*^st^*^+^ and VTA^Sst−^ mice

Behavioral testsfor VTA^S^*^st^*^+^ and VTA^Sst−^mice							
Name of test	Environment	Group size				Affected?	Statistical significance
		♂ Control	♂ Caspase	♁ Control	♁ Caspase		
Running wheel	Home cage	9	6	8	5	No	
Open field	Open arena	10	9	11	8	No	
Light/dark box	Test system	10	9	11	8	No	
Elevated plus maze	Test system	10	9	11	8	No	
Novelty suppresed feeding	Open arena	10	9	11	8	No	Tendency to sex-treatment interaction; caspase males start eating faster than controls and caspase females later than controls.
Sucrose preference	Home cage	10	9	11	7	No	
**Morphine-induced** **locomotor sensitization**	Open arena	10	7	11	8	**Yes**	Expression of morphine sensitization; caspase mice display stronger moprhine-induced locomotor sensitization.
**Forced-swim test**	Test system	10	9	11	8	**Yes**	Caspase animals struggle longer than controls.
Saccharine preference	IntelliCage	7	7	7	5	No	
**Home cage activity** **in group**	IntelliCage	7	7	7	5	**Yes**	Females are more active in nose poking to water-containing doors than males; caspase females are more active than control females.
Delay discounting	IntelliCage	7	7	7	5	No*	Strong sex interaction; females are ready to wait longer for the saccharine than males.
Reward-related learning	IntelliCage	7	7	7	5	No	
Reward-related unlearning	IntelliCage	7	7	7	5	No	
Reward-related unlearning with punishment	IntelliCage	7	7	7	5	No	
Reward-related relearning	IntelliCage	7	7	7	5	No	
**Fear Conditioning (FC)**	Test system	6	7	5	5	**Yes**	Sex-treatment interaction; caspase males freeze less, and caspase females freeze more than controls.
**FC context test**	Test system	6	7	5	5	**Yes**	Sex-treatment interaction; caspase males freeze less, and caspase females freeze more than controls.
FC cue test	Test system	6	7	5	5	No	

Boldface indicates significantly affected behaviors due to VTA^Sst^ neurons deletion to simplify their identification. Asterisk indicates no difference due to VTA^Sst^ neurons deletion, but strong difference between sexes in the test performance.

10.1523/ENEURO.0149-23.2023.f5-1 Figure 5-1 Deletion of the VTA^Sst^ neurons with caspase 3-expressing virus. ***a***, Scheme of the bilateral intra-VTA viral injections to Sst-tdTomato (magenta) mice. The control group received only myr-eGFP virus diluted with dH_2_0 to adjust the final volume. ***b***, The graph shows an average number of GFP+ Sst+ cell bodies in the control and caspase-treated animals (*n* = 5 animals per group) depicted per bregma level (*x*-axis). ***c***, Example images of the mouse coronal VTA section from the control (left) and caspase group (right). The caspase image has almost no infected GFP+ cell bodies in the VTA region (white dashed outline), showing only sparse GFP+ neurite fragments of the ablated Sst+ neurons. ***d***, Experimental timeline of behavioral tests conducted in three cohorts. DD, Delay discounting; EPM, elevated plus maze; LDB, light/dark box; LOC, novelty-induced locomotor activity; MOR, morphine sensitization; SacPref, saccharine preference; SacAvoid, Saccharine avoidance; SacExtinc, saccharine extinction, SP, sucrose preference; RW, running wheel activity; W, week. Download Figure 5-1, TIF file.

10.1523/ENEURO.0149-23.2023.f5-2 Figure 5-2 Deletion of Sst+ neurons in the VTA had no effect on locomotor activity or anxiety-like behavior. ***a***, Locomotor activity in the open arena was not different between the VTA^Sst^-caspase and control mice (*F*_(1,34)_ = 0.265, *p* = 0.61). ***b***, The light/dark box test did not show any difference in percentage of time spent in the light compartment between the treatment groups (*F*_(1,34)_ = 1.750, *p* = 0.195; sex, *F*_(1,34)_ = 3.957, p = 0.055). ***c***, Similarly, the percentage of time spent in the open arm measured in the elevated plus maze test was not different (treatment, *F*_(1,34)_ = 0.072, p = 0.79). ***d***, Latency to start eating in a novel environment did not show a difference, albeit a marginal significance for sex-dependent effects was detected in the VTA^Sst^-caspase mice (treatment × sex, *F*_(1,34)_ = 3.862, *p* = 0.058). Data are shown as mean ± SEM. Download Figure 5-2, TIF file.

10.1523/ENEURO.0149-23.2023.f5-3 Figure 5-3 Deletion of VTA^Sst^ neurons did not affect circadian activity in the free-running wheel test (lights on 6:00–18:00). Activity of the control (black) and VTA^Sst^-caspase (blue) mice. There were no differences in the number of rotations between the treatment groups across 3 days (treatment, *F*_(1,24)_ = 0.202, *p* = 0.657; treatment × time, *F*_(67,1608)_ = 1.230, *p* = 0.278). Data are shown as mean ± SEM. Download Figure 5-3, TIF file.

10.1523/ENEURO.0149-23.2023.5-1 Table 5-1 Statistical table for all behavioral tests. Download Table 5-1, XLS file.

### Fear conditioning is affected differently in VTA^Sst−^ males and females

**Figure 6.**
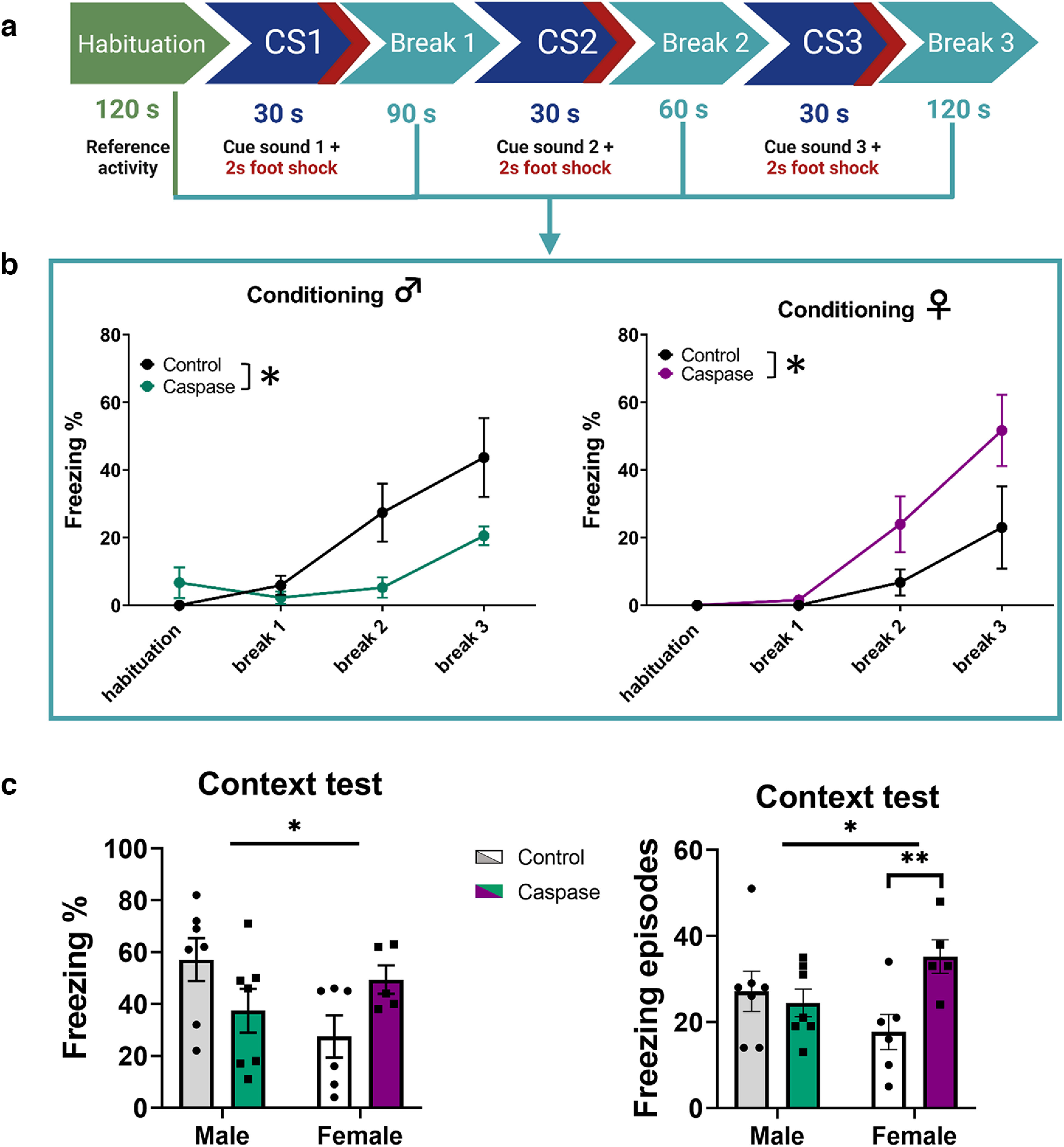
Deletion of VTA^Sst^ neurons affected fear conditioning in a sex-dependent manner. ***a***, Fear conditioning protocol during the acquisition phase (see above, Materials and Methods). ***b***, Graphs show percentage of freezing (freeze time/total time) during breaks following the conditioning episodes (sex × treatment, *F*_(1,21)_ = 9.971, *p* = 0.005). The VTA**^Sst^**-caspase males froze less during the breaks between footshocks, whereas the VTA**^Sst^**-caspase females, on the contrary, froze more (*post hoc* males, *p* = 0.036; females, *p* = 0.038). ***c***, A similar sex × treatment interaction (*F*_(1,21)_ = 6.602, *p* = 0.018 for freezing percentage; *F*_(1,21)_ = 6.102, *p* = 0.022 for freezing episodes) was observed in the context-associated fear memory retrieval test 9 d after the conditioning. Right, Graph shows that the VTA**^Sst^**-caspase females froze more often, but as seen in the left graph not significantly longer (freezing percentage) than the control females (freezing episodes, *post hoc* females, *p* = 0.01). Cue-induced fear-processing is shown in Extended Data Figure 6-1. Data are mean ± SEM; *p < 0.05, **p < 0.01.

Considering that VTA^Sst^ neurons mostly project to the brain areas that control response and memory formation to aversive events (Concetti et al., 2020; Goode and Maren, 2017; Keifer et al., 2015; Penzo et al., 2015), we further assessed possible changes in threat processing. We used a pavlovian fear conditioning paradigm (Maren, 2001), followed by contextual and cue-induced retrieval tests to assess differences in acquisition (acute response) and fear memory formation/expression. During the acquisition, mice were presented with three consecutive 30 s cue sounds coterminated with 2 s footshocks (0.6 mA) and separated with short breaks (Fig. 6*a*). Repeated-measures two-way ANOVAs for the percentage of freezing and number of the freezing episodes detected significant sex × treatment interactions (*p* = 0.004 and *p* = 0.035, respectively, Extended Data Table 5-1), indicating that the deletion of VTA^Sst^ neurons influenced reaction to aversive footshock stimuli in a sex-dependent manner. A deeper analysis of the data showed that this difference came exclusively from the time points between the footshocks when the sound was absent (breaks 1, 2 and 3; Fig. 6; Extended Data Fig. 6-1*b*). Indeed, *post hoc* analysis confirmed that the VTA^Sst−^ males froze less than the control males (*p* = 0.036) during the breaks between footshocks, whereas the VTA^Sst−^ females froze more than the control females (*p* = 0.038; Fig. 6*b*). Interestingly, there were no differences between the treatment groups when the cue sound was on (Extended Data Fig. 6-1*b*).

10.1523/ENEURO.0149-23.2023.f6-1 Figure 6-1 Deletion of the VTA^Sst^ neurons did not affect cue-induced fear processing in pavlovian fear conditioning. ***a***, Protocol of fear conditioning during the acquisition phase. ***b***, Graphs show percentage of freezing (freeze time/total time) during 30 s cue-sound presentations, coterminated with 2 s footshocks. There was no difference in freezing between sexes (*F*_(1,19)_ = 0.019, *p* = 0.891) or between treatments (*F*_(1,19)_ = 2.182, p = 0.156) . ***c***, Similarly, there were no significant differences in rates of cue-associated fear memory retrieval or extinction (cue × sex × treatment, *F*_(1,420)_ = 1.04, *p* = 0.413). Data are shown as mean ± SEM. Download Figure 6-1, TIF file.

Further, we tested context-induced fear memory retrieval by placing the mice for 5 min in the same chamber where they had received footshocks 9 d earlier. Again, there was a similar sex × treatment interaction (*p* = 0.018) in the percentage of freezing and freezing episodes (*p* = 0.022; Fig. 6*c*) as we saw during the conditioning. The *post hoc* test showed that the VTA^Sst−^ females had more freezing episodes compared with control females (*p* = 0.01; Fig. 6*c*). Cue-induced fear memory retrieval or extinction after repeated cue presentations was not significantly affected by the deletion of VTA^Sst^ neurons, although the results showed similar sex-dependent trends as during the conditioning and context testing Extended Data Fig. 6-1*c*).

### Deletion of VTA^Sst^ neurons delayed the onset of immobility in the forced-swim test

**Figure 7.**
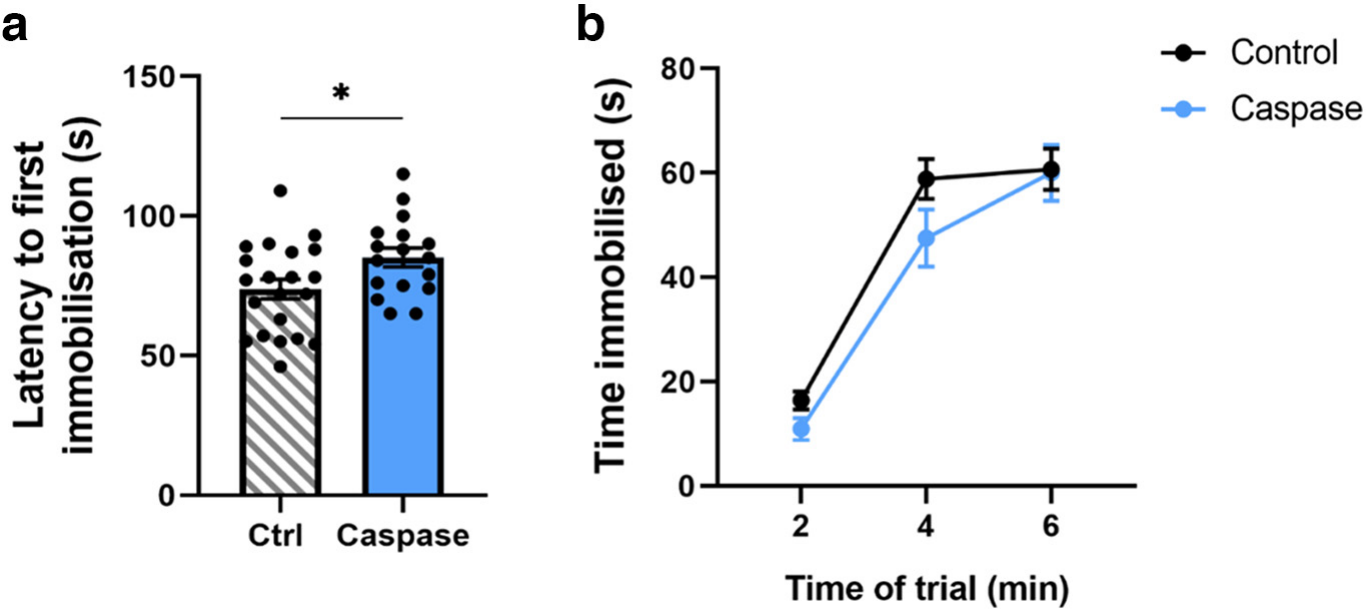
Delayed onset of immobility in the forced-swim test in the VTA^Sst^-caspase mice. ***a***, ***b***, VTA**^Sst^**-caspase mice showed a longer latency to the first immobilization event (***a***; *F*_(1,34)_ = 5.08, *p* = 0.031) and (***b***) a tendency for a shortened duration of immobilization (time × treatment, *F*_(2,68)_ = 1.938, *p* = 0.16) especially in the first 4 min. Data are mean ± SEM; dots in ***a*** show individual data points; *p < 0.05.

One of the sex-independent changes in the behavioral performance of the mice lacking VTA^Sst^ neurons was a delayed latency to the first immobility event in the forced-swim test (FST; Fig. 7). Results showed that the VTA^Sst−^ mice struggled longer than the control group (*p* = 0.031) and tended to spend less time immobile during the first 4 min of the test. As the tested animals were not exposed to any chronic stressor before the FST, the observed behavioral alteration might predominantly be related to a reaction to the acute unpredictable stressor.

### Deletion of VTA^Sst^ neurons affected morphine sensitization but not natural reward-related processing

**Figure 8.**
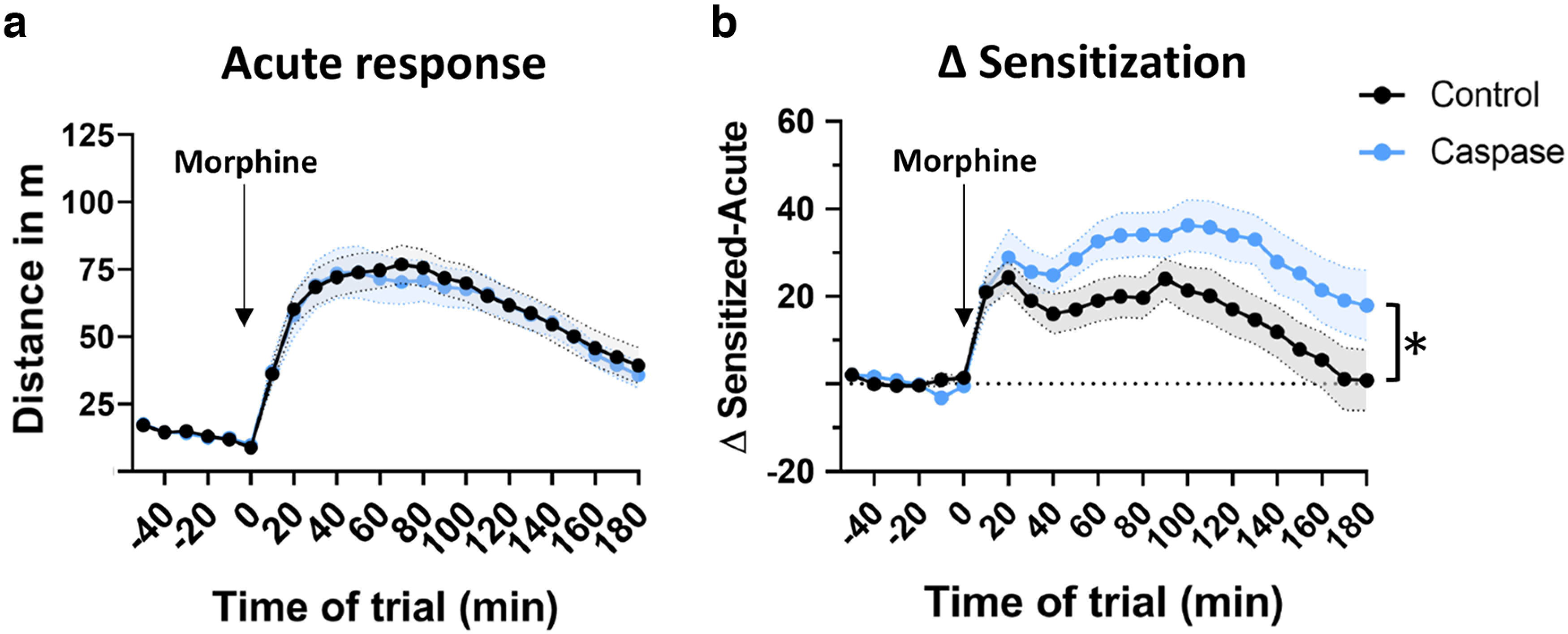
Increased morphine-induced locomotor sensitization in VTA^Sst−^ mice. ***a***, ***b***, Arrows indicate the time of morphine administration (20 mg/kg, i.p.) Acute treatment with morphine (***a***) induced a similar hyperlocomotor response in both treatment groups (treatment, *F*_(1,32)_ = 0.034, *p* = 0.854). Seven days after the first morphine injection (***b***), the response to the morphine challenge was enhanced and prolonged in the VTA**^Sst^**-caspase mice compared with controls (treatment, *F*_(1,32)_ = 12.014, *p* = 0.002; time × treatment, *F*_(23,735)_ = 2.915, *p* = 0.024). Responses to natural, sweet rewards are shown in Extended Data Figures 8-1 and 8-2. Data are shown across 10 min time bins as mean ± SEM; *p < 0.05.

Taking into account the previously shown ability of VTA^Sst^ neurons to inhibit neighboring DA cells (Nagaeva et al., 2020), it was important to find out whether drug and natural reward processing would be affected in VTA^Sst−^ animals. We chose morphine as the experimental substance as its rewarding potential is well known in rodents (Kuzmin et al., 1996; Martin et al., 1963), and its mechanism of action includes inhibition of VTA GABA cells resulting in excessive DA neuron firing by disinhibition (Johnson and North, 1992). There were no differences in the locomotor response to a single dose (20 mg/kg, i.p.) of morphine between the treatment groups (Fig. 8*a*), indicating that acute reaction to morphine was not affected in VTA^Sst−^ mice. However, we detected a significant enhancement in sensitization to locomotor activation by the second challenge dose (20 mg/kg, i.p.) of morphine in the VTA^Sst−^ mice as compared with the control mice (*p* = 0.002), 7 d after the first morphine injection (Fig. 8*b*).

10.1523/ENEURO.0149-23.2023.f8-1 Figure 8-1 Deletion of the VTA^Sst^ neurons did not affect natural reward preference or sensitivity. ***a***, Simplified scheme depicting corners (yellow) and side assignment of the saccharine preference test in the IntelliCage system. S1, 0.01% saccharine; S2, 0.03% saccharine; S3, 0.3% saccharine; W, water. ***b***, Graphs show preference in percentages (number of licks to a certain bottle/number of total licks, *y*-axis) to different saccharine concentrations over water (*x*-axis). Preference to different saccharine concentrations or water in the corresponding corner in the IntelliCage system did not reveal any significant differences between the treatment groups for low saccharine concentrations (*F*_(1,24)_ = 0.698, *p* = 0.413) or to high ones (*F*_(1,24)_ = 0.045, *p* = 0.834). The dashed line shows a 25% preference rate. ***c***, Similarly, sucrose preference in the two-bottle choice test in IVC cages did not reveal any differences (concentration × treatment, *F*_(2,66)_ = 0.362, *p* = 0.688). The dashed line shows a 50% preference rate. Data are shown as mean ± SEM. Download Figure 8-1, TIF file.

10.1523/ENEURO.0149-23.2023.f8-2 Figure 8-2 Relearning new rules in control and caspase mice and introduction of air puffs in the reward-related task. The *x*-axis shows the number of nose pokes to the saccharine bottles per hour, *y*-axis shows daily hours (lights on 6:00–18:00). ***a***, Nose-poking dynamics in male mice. Although there was a clear tendency in VTA^Sst^-caspase male mice to be less active in nose poking to the saccharine corner in all phases of the relearning-avoidance test, no statistically significant differences were detected between the groups (Extended Data Table 5-1). ***b***, Nose-poking dynamics in female mice showed no differences between the groups. Data are shown as mean ± SEM. Download Figure 8-2, TIF file.

As the natural reward to be tested, we chose sweetened drinking water but did not find any significant differences between the treatment groups in sucrose or saccharine consumption or preference over plain water (Extended Data Fig. 8-1). We also designed two reward-based learning tasks for the IntelliCage system aiming to assess alterations in prediction error processing (Schultz et al., 1997) and in punishment-resistant reward preference. For these tasks, the mice learned first to nose poke in assigned corners to receive access to 0.3% saccharine in water. Each nose poke to the saccharine door activated a light indicating that the saccharine would be available in 2 s. On the third day, when the task performance was stable, we either emptied the saccharine bottles or introduced 0.2 bar air puffs with a 25% probability at the end of the licking session (Radwanska and Kaczmarek, 2012). It is important to note that the two experimental tasks took place consecutively in the IntelliCage allowing assessment of the relearning rates after failed reward accesses.

The prediction error probing showed significant sex differences (*p* < 0.001) and sex × treatment interaction (*p* = 0.043) between the control and VTA^Sst−^ groups (data not shown). However, further *post hoc* analysis separately for males and females did not show any significant differences between the treatments, only showing a tendency for the VTA^Sst−^ females to relearn slower not to nose poke anymore in an emptied saccharine bottle (*p* = 0.064). For the next punishment-resistant reward preference test, saccharine was reintroduced in new corners after being unavailable for 2 d, and the mice had to relearn the rules. Although there was a tendency of the VTA^Sst−^ male mice to be less active in nose poking to saccharine corners in all phases of the relearning/avoidance test (Extended Data Fig. 8-2), we could not detect any statistically significant differences between the groups (Extended Data Table 5-1). Similarly, we did not detect any differences in reactions to air puffs.

### Delay discounting test revealed a sex-dependent but not VTA^Sst^ neuron-dependent difference

**Figure 9.**
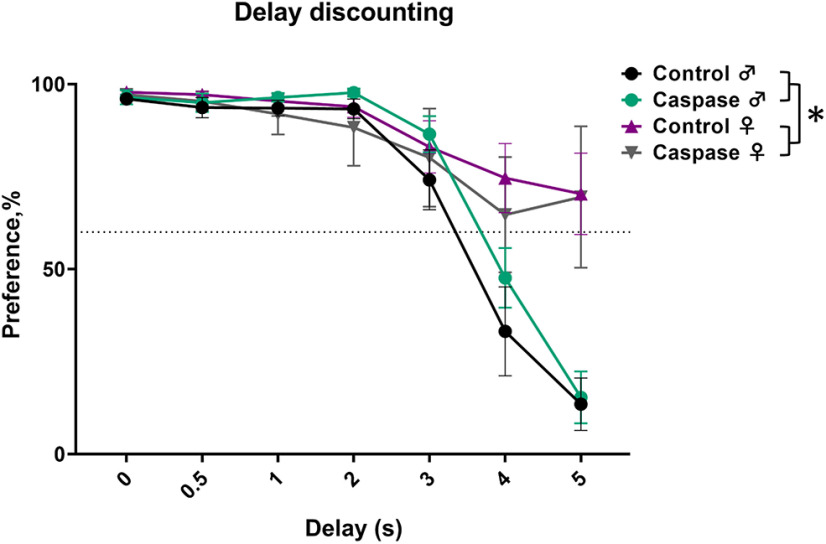
Deletion of the VTA^Sst^ neurons did not influence the impulsivity or readiness to wait for the saccharine reward. The *y*-axis indicates preference in percentage for 0.3% saccharine over water defined as a lick number to the saccharine bottle/total number of licks. The *x*-axis indicates the duration of the delay before the saccharine door opened after a mouse entered the corner. The asterisk indicates the significance of the differences between the sexes (*F*_(1,22)_ = 5.829, *p* = 0.024) and of the sex × delay interaction (*F*_(6,132)_ = 17.09, *p* < 0.0001). Data are shown as mean ± SEM. *p < 0.05.

Although we did not find any differences in the preference for saccharine or sucrose of various concentrations between the VTA^Sst−^ and control groups (Extended Data Fig. 8-1), we observed an interesting sex-dependent behavior in the delay discounting task (Mitchell, 2014) in the IntelliCage system. The delay discounting test, where mice learn to wait for a reward for increasing periods of time, showed a sex difference (*p* = 0.0239) with significant dependence on the duration of the delay (sex × delay interaction, *p* < 0.001; Extended Data Table 5-1). As shown in Figure 9, for both male groups the longer delay to the saccharine delivery drastically decreased the number of licks to the saccharine bottle starting from the 4 s delay and dropped almost to nonexistent at the 5 s delay, whereas females were still willing to wait and lick. The delay discounting test is usually interpreted as a measure of impulsivity, making male mice in our experiment more impulsive or less patient. However, we did not see any differences between the treatment groups within the sexes, suggesting that the deletion of VTA^Sst^ neurons did not affect impulsivity or readiness to wait for the reward.

## Discussion

In the present study, we demonstrated that VTA^Sst^ neurons, in addition to their previously shown local inhibitory activity onto neighboring DA cells (Nagaeva et al., 2020), consistently project to five forebrain areas: alBNST, CeM, PVT, LH and VP. Interestingly, these areas have been reported to also share Sst+ projections among each other, often reciprocally (Ahrens et al., 2018; Bartonjo and Lundy, 2022; Elsilä et al., 2018; Luo et al., 2018; Penzo et al., 2014; Sun et al., 2022; Yu et al., 2022). For example, in addition to the VTA (Yu et al., 2022), the BNST (Elsilä et al., 2018), CeA (Bartonjo and Lundy, 2022), and medial paralemniscus nucleus (Sun et al., 2022) send their Sst+ projection to the LH. The LH, in turn, innervates the CeA, BNST, and PVT (Luo et al., 2018) via its Sst+ axons. Altogether, this suggests the existence of a large subcortical Sst+ neuron network of which VTA^Sst^ neurons constitute a part. At the same time, it raises the question of the role of Sst expression in these projecting neurons. Previous work on neuroblastoma-derived cell culture reported higher neurite growth in cells that coexpressed SST and neuron-specific III β-tubulin compared with cells lacking this coexpression (Paik et al., 2019), suggesting that SST may play a role in organizing cytoskeleton proteins and is important for neurite formation and growth.

It is important to note that in the present study, similarly to the majority of the reports mentioned above, the *Sst* gene was predominantly used as the marker of the certain GABA or Glu neuronal subpopulations, and the functional role of the SST peptide itself was not addressed. As persistent depolarization of the neuron and recruitment of the dense core vesicles (van den Pol, 2012) are needed for the corelease of slow neuropeptide neurotransmitters with more easily released fast neurotransmitters (e.g., GABA or Glu), it remains to be determined whether the behavioral tests we used would provoke such intense activation of VTA^Sst^ neurons.

Projecting Sst+ neurons were found to have a specific location in the anterolateral part of the VTA, where most of the Sst+ neurons of the ADP subtype are located (Nagaeva et al., 2020). As expected, our patch-clamp experiments confirmed the ADP electrophysiological subtype for the backtraced VTA^Sst^ projection neurons. Given that ADP neurons are molecularly diverse, encompassing GABAergic and mixed phenotypes (Nagaeva et al., 2020), additional molecular and physiological investigations are required to determine the neurotransmitter characteristics of these cells. Specifically, it is crucial to ascertain whether VTA^Sst^ neurons communicate inhibitory or excitatory signals to their respective projection areas and identify the specific neuronal subtype they target in those areas. Acquiring this knowledge would greatly enhance our understanding of VTA^Sst^ circuits and their specific functions.

Our behavioral assessment of the mice with VTA^Sst^ neuron deletion aimed to screen for the possible roles of VTA^Sst^ neurons and importantly revealed the following four major consequences: increased home-cage activity, altered response in fear conditioning, enhanced locomotor sensitization to morphine, and prolonged struggle during the inescapable stress of forced swimming. The first two changes showed a significant sexual dimorphism.

Regarding the home-cage activity, it is well known that females are more active than males in the IntelliCage environment where they are group housed (Pernold et al., 2021, 2019), which we also observed (Extended Data Table 5-1). Interestingly, the deletion of VTA^Sst^ neurons resulted in a further increase of the home-cage activity exclusively in the female mice (Fig. 5). The activity change was not associated with the novelty of the environment as it was consistent throughout the 3 d experiment. Circadian patterns in single-housed animals determined during 3 d access to free-running wheels failed to differ between VTA^Sst−^ and control animals (Extended Data Fig. 5-3). In addition, there was no change in locomotor activity in a novel environment as the distance traveled in the open field was similar between the two treatment groups. Our data suggest that the deletion of VTA^Sst^ neurons resulted in a specific increase in home-cage activity and only in group-housed female mice.

Although several VTA^Sst^ neuron projection targets, like the BNST (Lebow and Chen, 2016), CeM (Shackman and Fox, 2016), and PVT (Kirouac, 2021), are involved in the brain anxiety network, we did not see any differences in anxiety-like behaviors in VTA^Sst−^ mice (Extended Data Figs. 5-2, 8–2), similar to a previous report (Yu et al., 2022). Instead, our data indicate that the VTA^Sst^ neurons could regulate responses to inescapable acute stressors, such as freezing after unexpected footshock (fear-conditioning acquisition phase) or struggling when dipped into water (forced-swim test).

An interesting effect of the deletion of VTA^Sst^ neurons was the opposite change in the reaction to the footshock between males and females (Fig. 6*b*). It is important to note that our data do not exclude the possibility that the nociceptive reaction itself was altered in the VTA^Sst−^ animals explaining why VTA^Sst−^ males froze less and VTA^Sst−^ females froze more than controls in response to the footshock. Interestingly, a recent study described a pathway from the laterodorsal tegmentum via the VTA to the basolateral amygdala, the inhibition of which reduced unconditioned freezing response to footshocks in male mice, similar to what we observed in the VTA^Sst−^ males (Broussot et al., 2022). Unfortunately, results on females were not reported in that study. Our data emphasize the importance of conducting experiments on both sexes, especially in case of reactions to aversive stimuli (Cover et al., 2014). The fear-conditioning test highlighted another feature of VTA^Sst^ neuron function, that is, these neurons appeared important for the contextual fear memory formation/retrieval but not for the sound-cue-associated responses, likely relating to differential processing of these two sensory modalities of conditioned stimuli.

The fact that many brain regions receiving VTA^Sst^ neuron projections belong to the extended amygdala suggests the involvement of these neurons in the action of addictive substances, such as opioids (Koob et al., 2014; Korpi et al., 2015). We did not see any differences in locomotor responses to acute morphine administration (Fig. 8*a*), indicating that the stimulating effect of an opioid was not affected by the deletion of VTA^Sst^ neurons and that opioid-induced disinhibition of DA neurons (Johnson and North, 1992) was probably not mediated by the local inhibition of VTA^Sst^ neurons. This is consistent with the data on rats, where morphine actions on VTA DA neurons could be prevented by silencing GABA neurons in the rostromedial tegmental nucleus (also called the tail of VTA; Jalabert et al., 2011), which might be more meaningful for the acute disinhibitory action of opioids on VTA DA neurons. More importantly, we did observe that after the second morphine administration 7 d later, the VTA^Sst−^ mice of both sexes demonstrated robustly increased locomotor sensitization, suggesting that VTA^Sst^ neurons normally limit the sensitization to opioids while not affecting acute effects of opioids. This might be linked to the morphine-induced adaptation of GABA_A_ receptor-mediated transmission onto VTA DA neurons (Nugent et al., 2007), which would be missing from intra-VTA synapses of Sst+ neurons in caspase-treated VTA^Sst−^ mice.

Interestingly, previous studies have shown that stressful events, including those of an emotional nature, often result in a similar increase in opioid sensitization leading to higher morphine preference (Kalivas et al., 1986; Kuzmin et al., 1996; Leyton and Stewart, 1990; Shaham et al., 1992). As it has been shown that the deletion of VTA^Sst^ neurons disrupts normal restorative sleep after social defeat stress (Yu et al., 2022), we can speculate that in both cases VTA^Sst^ neurons are involved in adaptive changes needed to restore the VTA circuit to the baseline state. The fact that VTA^Sst^ neurons appear to have protective properties in social stress and morphine sensitization warrants further studies on whether these cells can affect development of opioid addiction.

In summary, our study has demonstrated that in addition to their traditional role as local interneurons, VTA^Sst^ neurons can send long-range projections outside the hosting region and innervate several forebrain areas. Specifically, the VTA connection with the alBNST and PVT through Sst+ neurons was shown here for the first time. These findings expand the knowledge of efferent pathways from the VTA and also add VTA^Sst^ neurons to an emerging subcortical network of Sst+-projecting neurons. Moreover, our extensive behavioral screening revealed that VTA^Sst^ neurons are involved in the reactions to different stressors and morphine sensitization. Future research using optogenetics or chemogenetics should aim to dissect whether VTA^Sst^ neurons contribute to the observed behaviors through their projecting targets or by affecting the microcircuitry within the VTA. Taking into account that the VTA harbors a high percentage of neurons with mixed neurotransmitter phenotypes (Bouarab et al., 2019; Li et al., 2013; Miranda-Barrientos et al., 2021; Yamaguchi et al., 2015), confirming transmitter identities of the separate VTA Sst-expressing neuronal projections will be crucial to further understand how the reported behaviors are mediated mechanistically.

## Synthesis

Reviewing Editor: Tod E Kippin, University of California Berkeley Decisions are customarily a result of the Reviewing Editor and the peer reviewers coming together and discussing their recommendations until a consensus is reached. When revisions are invited, a fact-based synthesis statement explaining their decision and outlining what is needed to prepare a revision will be listed below. The following reviewer(s) agreed to reveal their identity: Ilia Karatsoreos, William Wisden. Note: If this manuscript was transferred from JNeurosci and a decision was made to accept the manuscript without peer review, a brief statement to this effect will instead be what is listed below.

I have received two independent reviews of your manuscript and both indicate that the paper makes important contributions to the field but also that some minor revisions are necessary prior to a publication decision can be made. Below are specific comments by the reviewers; please address all comments in preparing a revised submission.

Reviewer#1

This paper provides compelling evidence for a novel population of VTA SST+ neurons that are NOT just interneurons and, indeed, project more widely to several forebrain areas. The Authors also provide many converging lines of experiments that phenotype these cells, and highlight the their roles (or not) in a wide array of behaviors. This includes some loss of function experiments which add to the overall strengths of the manuscript. While the manuscript on its own does not serve as a particularly groundbreaking finding in regards to the overall field, it serve an important (and some would say critical) foundational role, opening up many new questions. In sum, many questions that I would have about the specific functional role of these neurons (via more direct causal experiments) I would not be able to formulate WITHOUT this paper.

I have several minor comments:

1) I would like a bit more discussion about how the authors think this fits into existing literature, and how knowing these specific new findings position the field to make gains.

2) I would ask the authors to be more consistent with the placement of scale bars in all of their photomicrographs, and making clear what the scales are. There are several figures that do not have any scale bars (e.g. Fig. 2, some supplemental figures) while others do (e.g. Fig. 3).

3) A visual abstract would be very helpful given the significant amount of data in this paper, some null some positive, and the lack of clarity on the overall significance.

The authors should be applauded for doing such a wide characterization, including some loss of function experiments.

Reviewer#2

The VTA is a key node involved in arousal, stress, motivation, addiction etc. This brain area contains complex networks of glutamate and GABA neurons, as well as the more studied dopamine neurons. This is a nice study from Nageeva and colleagues focussing on somatostatin neurons in the VTA, how they project, and what behaviours they contribute to. The male-female differences are also interesting. The paper will be useful to the enormous number of investigators looking at VTA circuitry and behaviour.

I had only relatively small comments:

1. Abstract. Line 29. Rather than write “innervate several forebrain regions” please write out what these regions are.

2. Line 37. What is meant by “making them a meaningful addition”? Do you mean making them relevant? Maybe rephrase?

3. Figure 4 Change the title from “Electrophysiological and expression profiles of VTA neurons projecting to the forebrain” to “Electrophysiological and expression profiles of VTA Sst neurons projecting to the forebrain”. Or even better “Electrophysiological profiles of VTA Sst neurons projecting to the forebrain” (see next point).

4. The qPCR results presented in Figure 4 part C for the Sst-Cre neurons are not ideal. As the authors point out, many of the neurons have undetectable sst expression even though they are supposed to be sst cells. It could be, as the authors say, that the Sst-Cre mice have a knockdown of sst expression, in which case this decrease should be measured in control mice (perhaps in e.g. cortex or whole VTA punches); or alternatively, for whatever reason, the qPCR has not been reliable (even though EGFP is detected as a positive control in all cells). The huge gaps in sst expression (just 30% of cells) make it hard to judge how much should be read into any discussion (or mention) of the presence or absence of other markers such as VGlut2 or Th expression in these cells. I am also quite surprised only one of the sampled cells has vgat expression, given that they have been documented as being inhibitory cells. Without doing more controls, I recommend to simply delete the gene expression data (i.e., Fig. 4c) from the paper. It does not make any difference to the main conclusions of the paper about behaviour influenced by VTA sst cells and their projection targets.

5. For the caspase virus, the authors should also cite: Yang, C. F. et al. Sexually dimorphic neurons in the ventromedial hypothalamus govern mating in both sexes and aggression in males. Cell 153, 896-909 (2013). This paper is where the caspase technique was first published.

## Author Response

Dear Dr. Nagaeva:

I am pleased to inform you that your manuscript, “Somatostatin-expressing neurons in the ventral tegmental area innervate specific forebrain regions and are involved in stress response,” has been judged potentially suitable for publication in eNeuro, provided appropriate revisions are made. The decision was a result of the reviewers and me coming together and discussing our recommendations until a consensus was reached. I have put together a fact-based synthesis statement explaining the decision and appended it to this email.

It is important that you revise your manuscript to address the reviewers’ concerns and submit a point-by-point reply to each concern. Before making a final decision, I will carefully review your revisions and responses to the reviewers.

Your revision must include the manuscript with new text indicated in a bold or highlighted font to aid the re-review of the manuscript. Separately, please provide a clean copy of the manuscript that includes the title page, which will be published if accepted. Please closely review your manuscript at this time for any final corrections in style or substance. Please consult our Revised Submission Checklist for full details on preparing your revision: https://www.eneuro.org/content/revising-finalizing-manuscript#revisions

In an effort to improve rigor, transparency, and reproducibility, eNeuro strongly encourages the use of estimation statistics. If the use of estimation statistics applies to your research, you may consider including it in your revision. More details and resources can be found in the Information for Authors: https://www.eneuro.org/content/preparing-manuscript#statistics

Your submission must include publication-quality figures, each in a separate EPS or TIFF (300 dpi) file. Please make sure your figures adhere to style requirements to avoid delays in manuscript processing. Detailed guidelines for figures are available in our Information for Authors: https://www.eneuro.org/content/preparing-manuscript#figures

When uploading the revised manuscript, the corresponding author will need to complete the electronic License to Publish form; if not completed during submission, a link to the form will also be available on the author’s home page at https://eneuro.msubmit.net.

All authors are invited to submit visually appealing, high-quality images to be used to promote their article, including on the journal homepage. Please submit your images, as well as an image caption and credit information, directly to the peer review system with your revision. Additional details on sizing and image specifications can be found in the Information for Authors: https://www.eneuro.org/content/revising-finalizing-manuscript#image

Please return your revision within 3 months of this decision. When you are ready to submit your revision, you can log in using the link below and click on the manuscript number to create your revision. If you have any questions or concerns, or need more time to prepare your revision, please contact eneuro@sfn.org.

https://eneuro.msubmit.net/cgi-bin/main.plex

Yours sincerely,

Tod E Kippin

Reviewing Editor

eNeuro

---------------------------------------------

Manuscript Instructions

submit a revised manuscript

Please note that the order of the authors has been changed, and that there are two equally contributing first authors.

We added estimation statistics in the provided statistical extended table.

We modified figure 4 according to the reviewer’s comments and added there the clustering results, which previously were in the supplementary figure.

We renamed extended figures and tables according to eNeuro requirements.

---------------------------------------------

Synthesis of Reviews:

Computational Neuroscience Model Code Accessibility Comments for Author (Required):

N/A

Synthesis Statement for Author (Required):

I have received two independent reviews of your manuscript and both indicate that the paper makes important contributions to the field but also that some minor revisions are necessary prior to a publication decision can be made. Below are specific comments by the reviewers; please address all comments in preparing a revised submission.

Reviewer#1

This paper provides compelling evidence for a novel population of VTA SST+ neurons that are NOT just interneurons and, indeed, project more widely to several forebrain areas. The Authors also provide many converging lines of experiments that phenotype these cells, and highlight the their roles (or not) in a wide array of behaviors. This includes some loss of function experiments which add to the overall strengths of the manuscript. While the manuscript on its own does not serve as a particularly groundbreaking finding in regards to the overall field, it serve an important (and some would say critical) foundational role, opening up many new questions. In sum, many questions that I would have about the specific functional role of these neurons (via more direct causal experiments) I would not be able to formulate WITHOUT this paper.

I have several minor comments:

1) I would like a bit more discussion about how the authors think this fits into existing literature, and how knowing these specific new findings position the field to make gains.

- We have added additional discussion. Lines 711-716, 729-736 and 823-834. Marked in yellow.

2) I would ask the authors to be more consistent with the placement of scale bars in all of their photomicrographs, and making clear what the scales are. There are several figures that do not have any scale bars (e.g. Fig. 2, some supplemental figures) while others do (e.g. Fig. 3).

- Thank you for pointing this. Scale bars have been added to all photomicrographs.

3) A visual abstract would be very helpful given the significant amount of data in this paper, some null some positive, and the lack of clarity on the overall significance.

- Visual abstract has been added to the submission. We hope, it will help the readers to go through the article.

The authors should be applauded for doing such a wide characterization, including some loss of function experiments.

-Thank you!

Reviewer#2

The VTA is a key node involved in arousal, stress, motivation, addiction etc. This brain area contains complex networks of glutamate and GABA neurons, as well as the more studied dopamine neurons. This is a nice study from Nageeva and colleagues focussing on somatostatin neurons in the VTA, how they project, and what behaviours they contribute to. The male-female differences are also interesting. The paper will be useful to the enormous number of investigators looking at VTA circuitry and behaviour.

I had only relatively small comments:

1. Abstract. Line 29. Rather than write “innervate several forebrain regions” please write out what these regions are.

- Done. Lines 30-33.

2. Line 37. What is meant by “making them a meaningful addition”? Do you mean making them relevant? Maybe rephrase?

- Changed to “relevant addition”. We meant that Sst neurons in the VTA form efferent pathways, which are different from the previously known VTA efferents. Similarly, these VTA Sst pathways add to the existing stress-related circuits, as our behavioural assays suggest.

3. Figure 4 Change the title from “Electrophysiological and expression profiles of VTA neurons projecting to the forebrain” to “Electrophysiological and expression profiles of VTA Sst neurons projecting to the forebrain”. Or even better “Electrophysiological profiles of VTA Sst neurons projecting to the forebrain” (see next point).

- Done. And the heat map has been deleted.

4. The qPCR results presented in Figure 4 part C for the Sst-Cre neurons are not ideal. As the authors point out, many of the neurons have undetectable sst expression even though they are supposed to be sst cells. It could be, as the authors say, that the Sst-Cre mice have a knockdown of sst expression, in which case this decrease should be measured in control mice (perhaps in e.g. cortex or whole VTA punches); or alternatively, for whatever reason, the qPCR has not been reliable (even though EGFP is detected as a positive control in all cells). The huge gaps in sst expression (just 30% of cells) make it hard to judge how much should be read into any discussion (or mention) of the presence or absence of other markers such as VGlut2 or Th expression in these cells. I am also quite surprised only one of the sampled cells has vgat expression, given that they have been documented as being inhibitory cells. Without doing more controls, I recommend to simply delete the gene expression data (i.e., Fig. 4c) from the paper. It does not make any difference to the main conclusions of the paper about behaviour influenced by VTA sst cells and their projection targets.

- Thank you for your comment. Unfortunately, we are not able to do any extra experiments within the provided 3 months. Therefore, we decided to delete this piece of data from the current manuscript, as we agree that it would be useful to add more neurons and positive controls. However, in positive dopaminergic control neurons from Th-EGFP mice we did not see any false positives, showing that those neurons did not express neither Vglut2 nor Vgat. We hope to finish these experiments and publish the results in the following paper, as this piece took quite an effort and also includes valuable chemistry for the single-cell qPCR method.

5. For the caspase virus, the authors should also cite: Yang, C. F. et al. Sexually dimorphic neurons in the ventromedial hypothalamus govern mating in both sexes and aggression in males. Cell 153, 896-909 (2013). This paper is where the caspase technique was first published.

- Thank you! We added this important reference. Line 566.
