# Supplementary figures and images for: Somatostatin-Expressing Neurons in the Ventral Tegmental Area Innervate Specific Forebrain Regions and Are Involved in Stress Response
Source: eNeuro. 2023 Aug 28;10(8):ENEURO.0149-23.2023. doi: 10.1523/ENEURO.0149-23.2023 (PMC10464661; doi:10.1523/ENEURO.0149-23.2023)

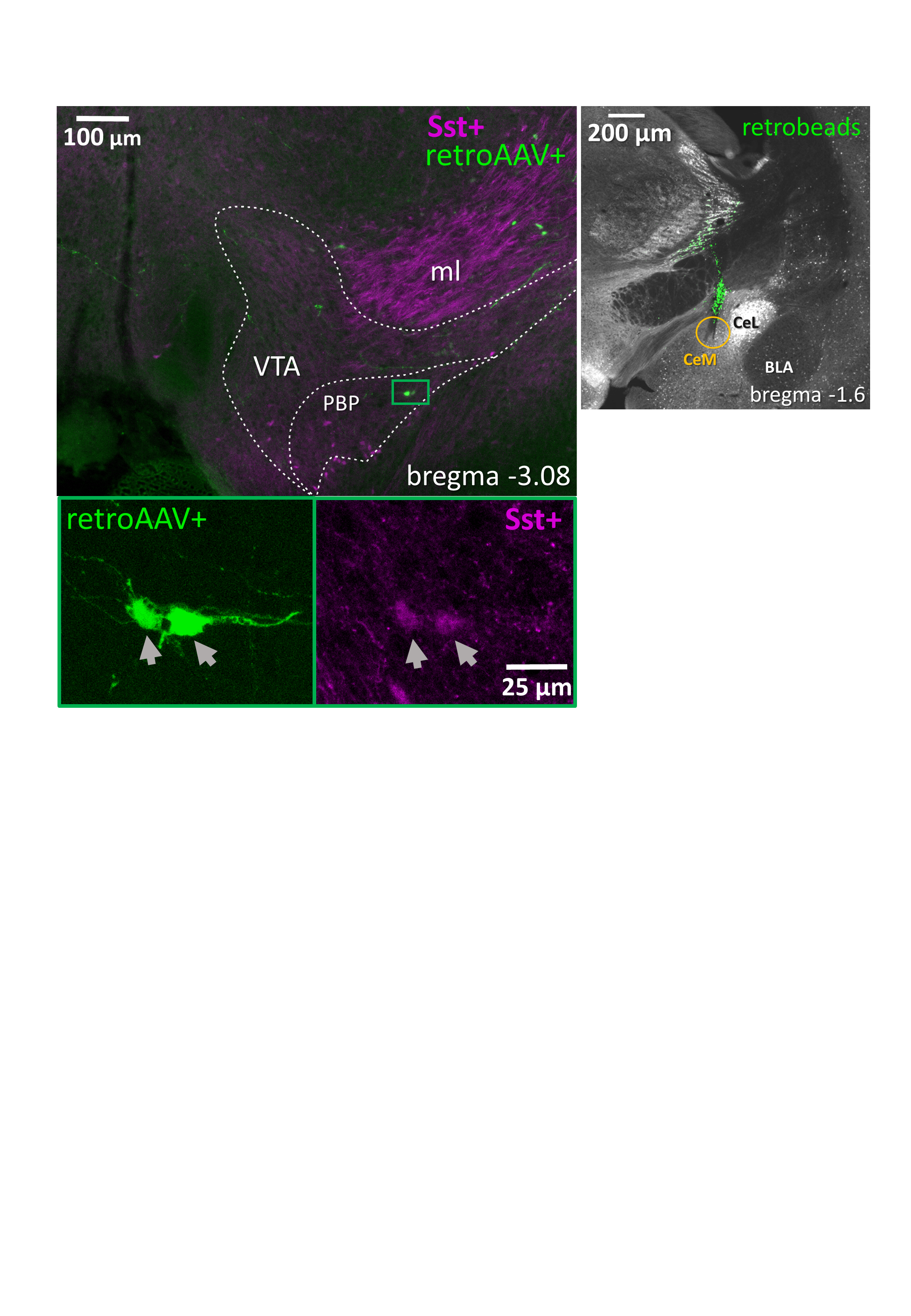

Supplement: Figure 3-1 — Backtracing from the medial part of the central amygdala. Examples of the backtraced Sst+ neurons in the VTA at the bregma level −3.08 mm in Sst-tdTomato (magenta) mouse. Right, Image shows RetroBeads in the injection site (CeM). The yellow circle shows the actual unilateral injection spot. Top left, Green rectangle shows ipsilaterally traced neurons. Bottom, Magnified images of the green rectangle. BLA, Basolateral amygdala; CeL, lateral part of the central amygdala; PBP, parabrachial pigmented nucleus of the VTA. Download Figure 3-1, TIF file. [file enu-eN-NWR-0149-23-s02.tif]

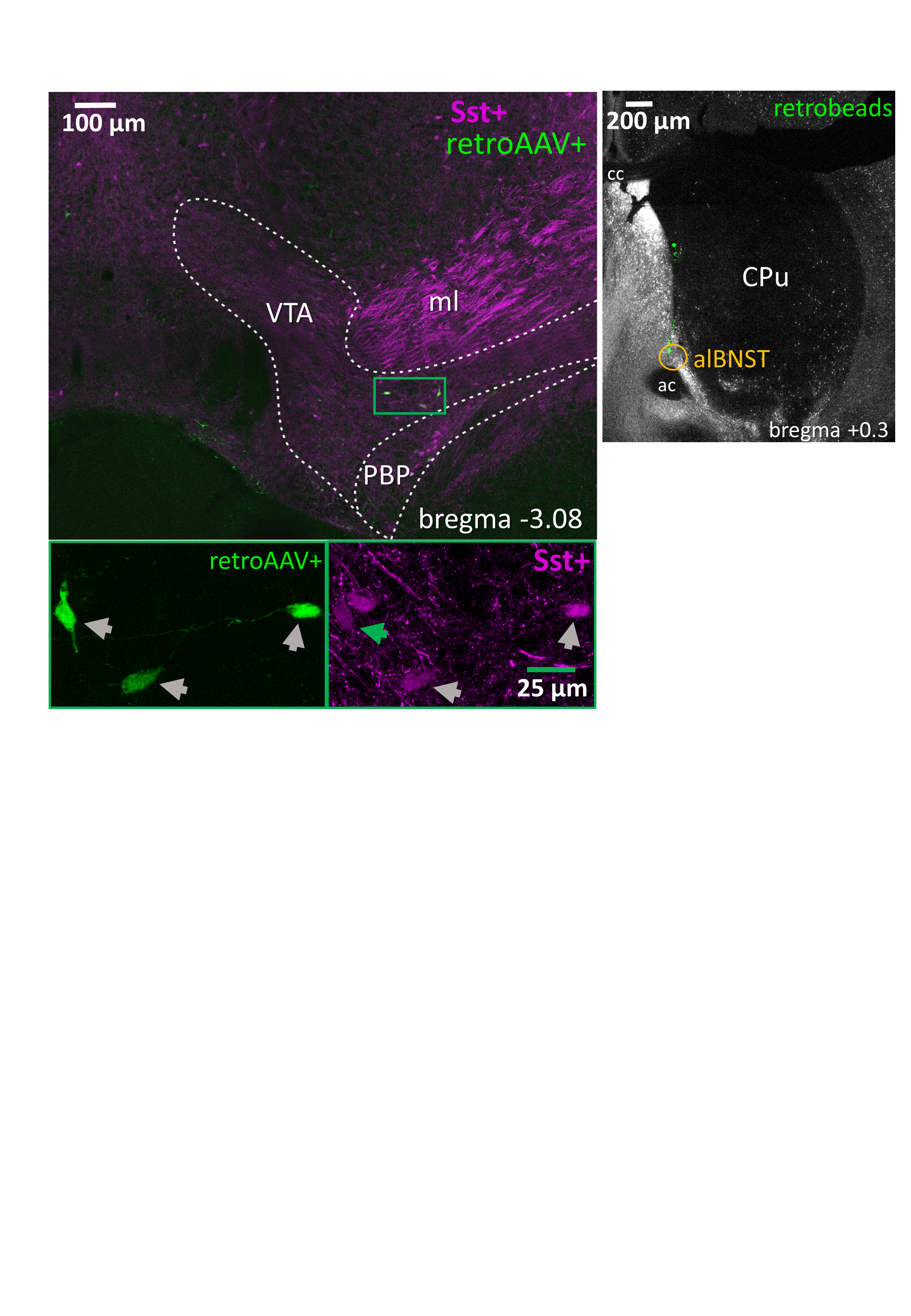

Supplement: Figure 3-2 — Backtracing from the anterolateral part of the bed nucleus of stria terminalis. Examples of the backtraced Sst+ neurons in the VTA at the bregma level −3.08 mm in Sst-tdTomato (magenta) mouse. Right, Image shows RetroBeads in the injection site (alBNST). The yellow circle shows the actual injection spot. Top left, Green rectangle shows ipsilaterally traced neurons. Bottom, Magnified images inside green rectangle split by fluorescent channels. ac, Anterior commissure; cc, corpus callosum; CPu, caudatus-putamen (striatum); ml, medial lemniscus; PBP, parabrachial pigmented nucleus of the VTA. Download Figure 3-2, TIF file. [file enu-eN-NWR-0149-23-s03.tif]

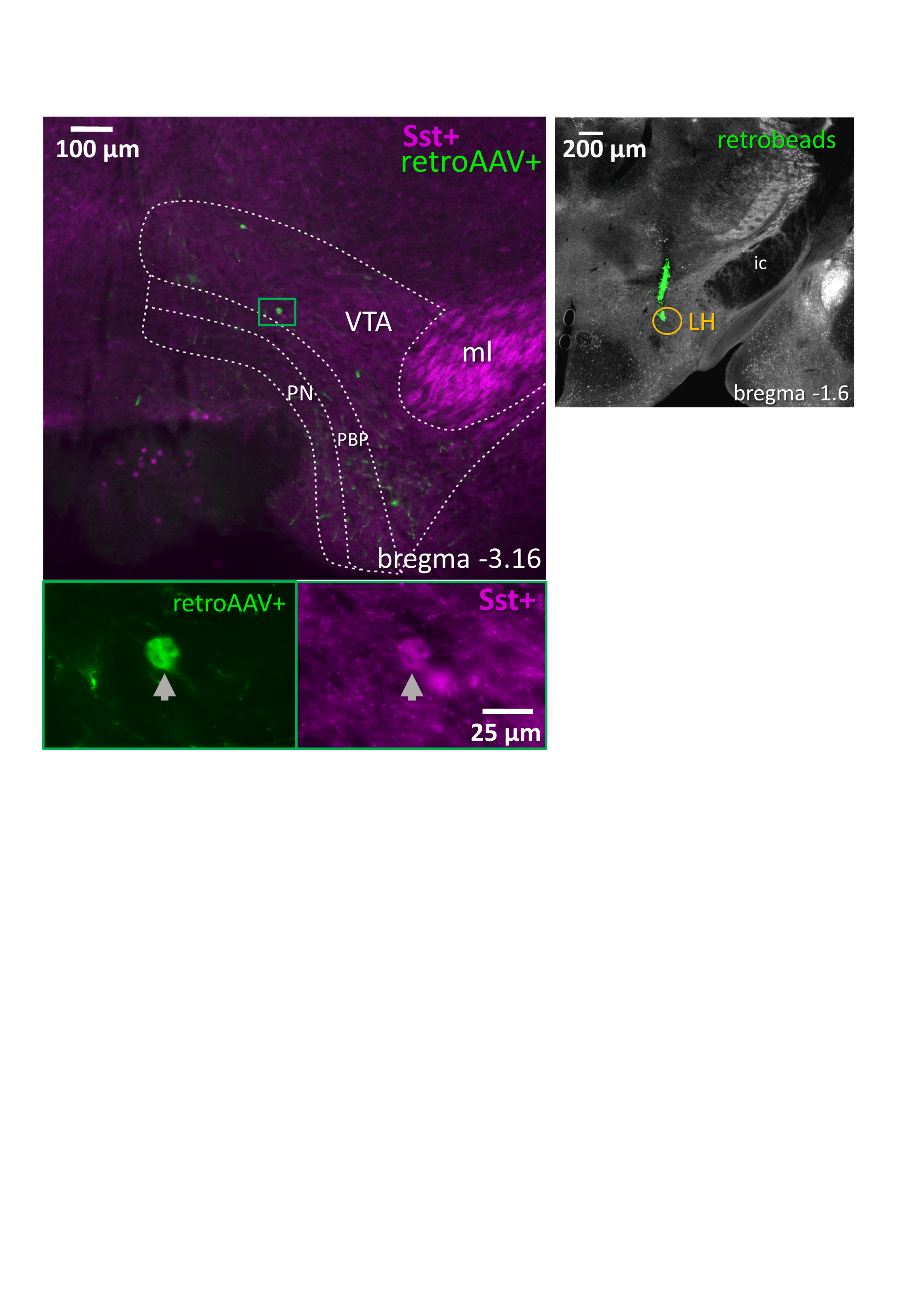

Supplement: Figure 3-3 — Backtracing from the lateral hypothalamus. Examples of the backtraced Sst+ neurons in the VTA at the bregma level –3.28 mm in Sst-tdTomato (magenta) mouse. Top right, RetroBeads at the injection site (LH). The yellow circle shows the actual unilateral injection spot. Top left, Green rectangle shows an ipsilaterally traced neuron. Bottom, Magnified images inside the green rectangle split by fluorescent channels. ic, Internal capsule; ml, medial lemniscus; PBP, parabrachial pigmented nucleus of the VTA; PN, paranigral nucleus of VTA. Download Figure 3-3, TIF file. [file enu-eN-NWR-0149-23-s04.tif]

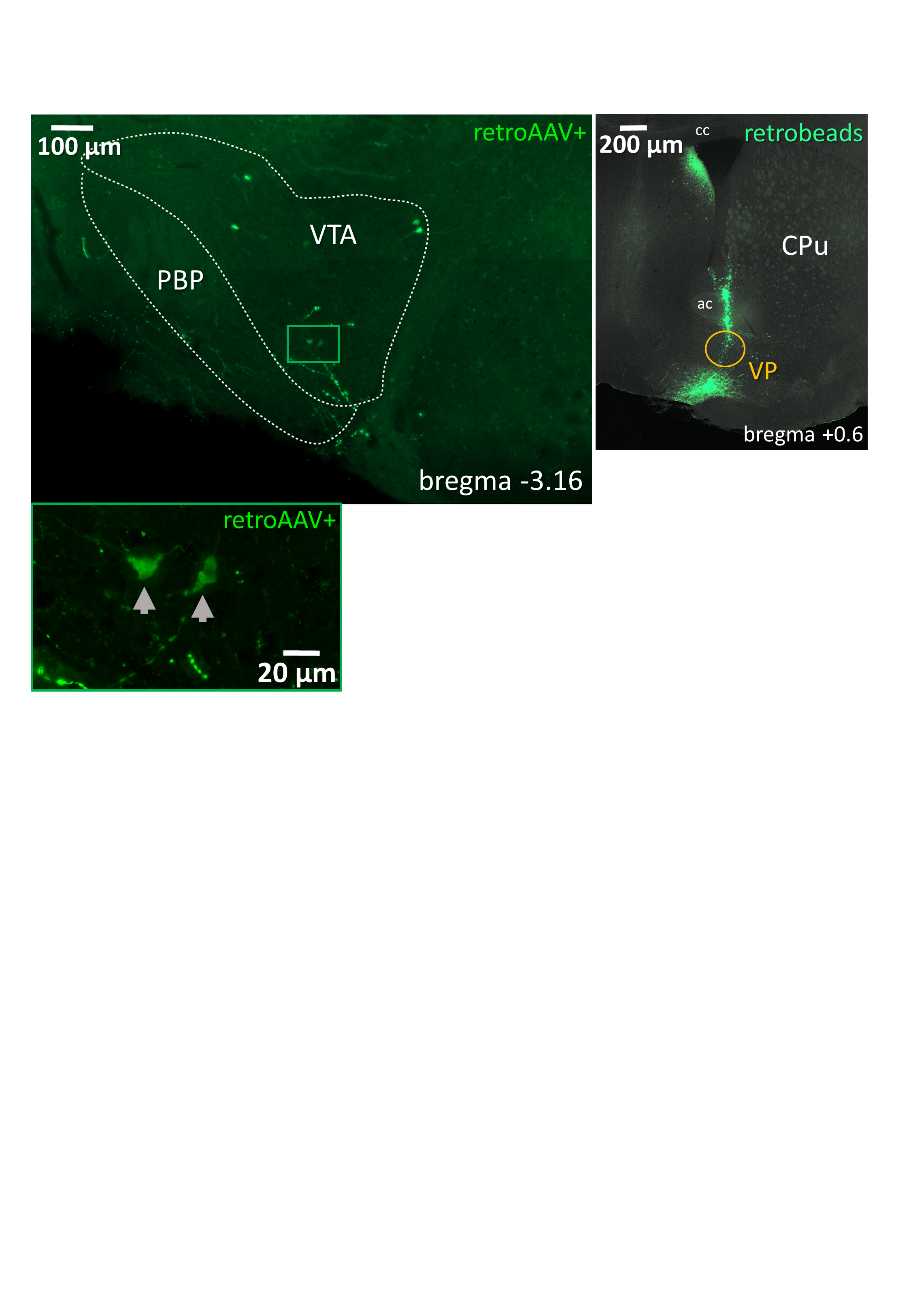

Supplement: Figure 3-4 — Backtracing from the ventral pallidum. Examples of the backtraced Sst+ neurons in the VTA at the bregma level −3.08 mm in Sst-Cre mouse. Right, The image shows RetroBeads in the injection site (VP). The yellow circle shows the actual unilateral injection spot. Top left, Green rectangle shows ipsilaterally traced neurons. Bottom, The magnified image of the green rectangle. ac, Anterior commissure; cc, corpus callosum; CPu, caudatus-putamen (striatum); PBP, parabrachial pigmented nucleus of the VTA. Download Figure 3-4, TIF file. [file enu-eN-NWR-0149-23-s05.tif]

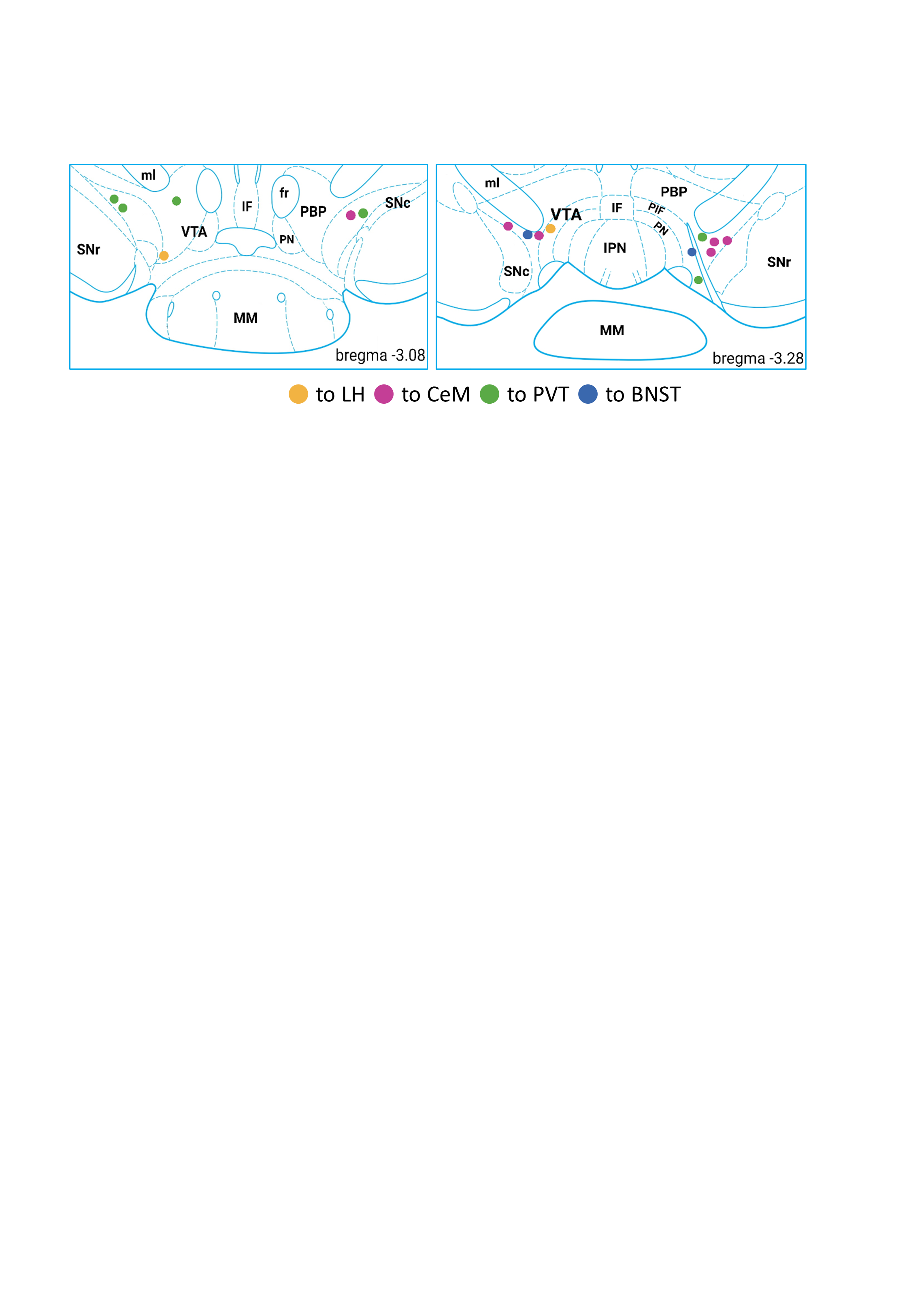

Supplement: Figure 4-1 — Location of the electrophysiologically recorded VTASst neurons projecting to forebrain regions and their electrophysiological subtypes. a, None of the backtraced neurons in electrophysiological experiments were found more posterior than the bregma level −3.28 mm, and most of them were located in the lateral nuclei of the VTA. Their projection sites are color coded. Download Figure 4-1, TIF file. [file enu-eN-NWR-0149-23-s06.tif]

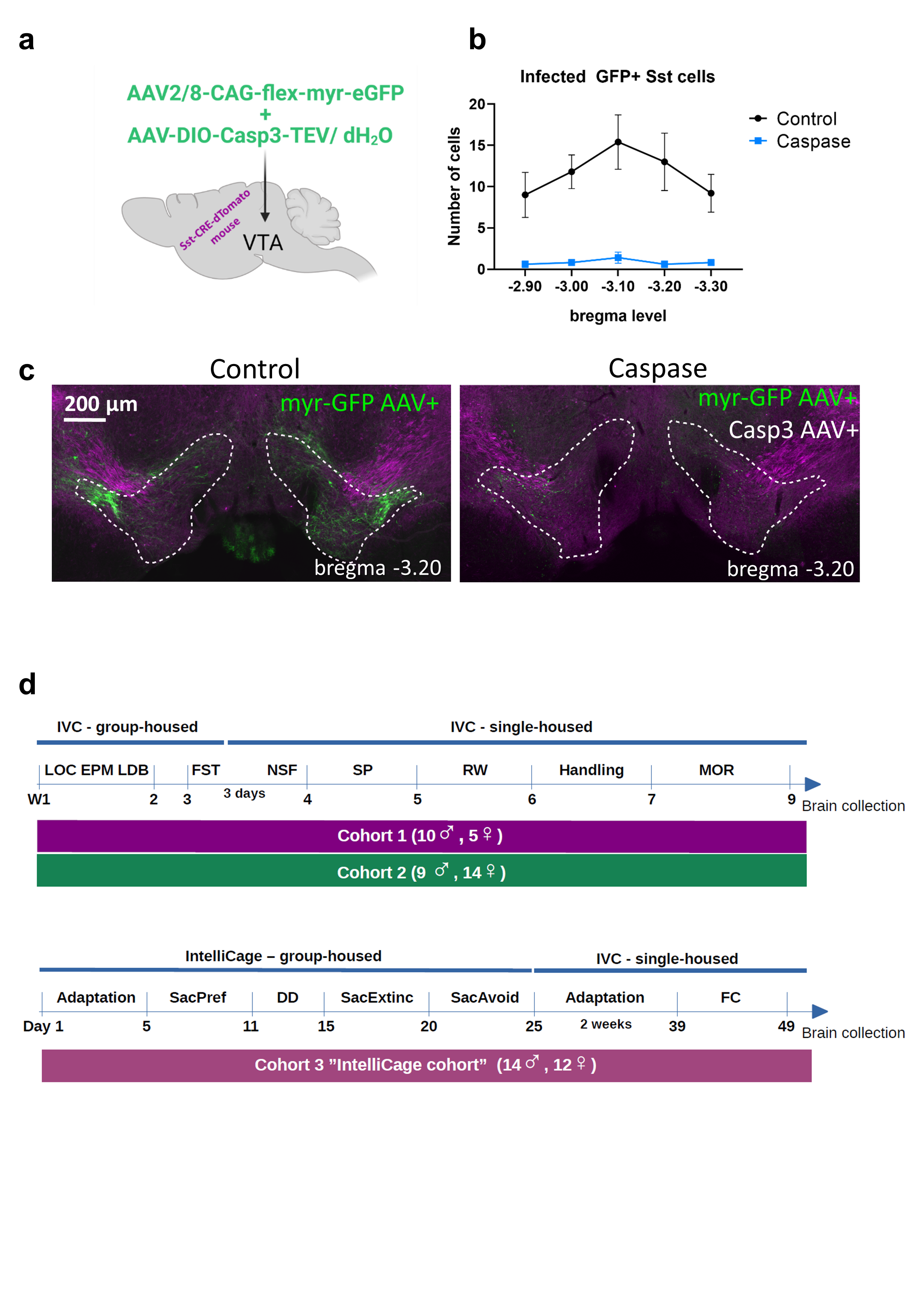

Supplement: Figure 5-1 — Deletion of the VTASst neurons with caspase 3-expressing virus. a, Scheme of the bilateral intra-VTA viral injections to Sst-tdTomato (magenta) mice. The control group received only myr-eGFP virus diluted with dH20 to adjust the final volume. b, The graph shows an average number of GFP+ Sst+ cell bodies in the control and caspase-treated animals (n = 5 animals per group) depicted per bregma level (x-axis). c, Example images of the mouse coronal VTA section from the control (left) and caspase group (right). The caspase image has almost no infected GFP+ cell bodies in the VTA region (white dashed outline), showing only sparse GFP+ neurite fragments of the ablated Sst+ neurons. d, Experimental timeline of behavioral tests conducted in three cohorts. DD, Delay discounting; EPM, elevated plus maze; LDB, light/dark box; LOC, novelty-induced locomotor activity; MOR, morphine sensitization; SacPref, saccharine preference; SacAvoid, Saccharine avoidance; SacExtinc, saccharine extinction, SP, sucrose preference; RW, running wheel activity; W, week. Download Figure 5-1, TIF file. [file enu-eN-NWR-0149-23-s07.tif]

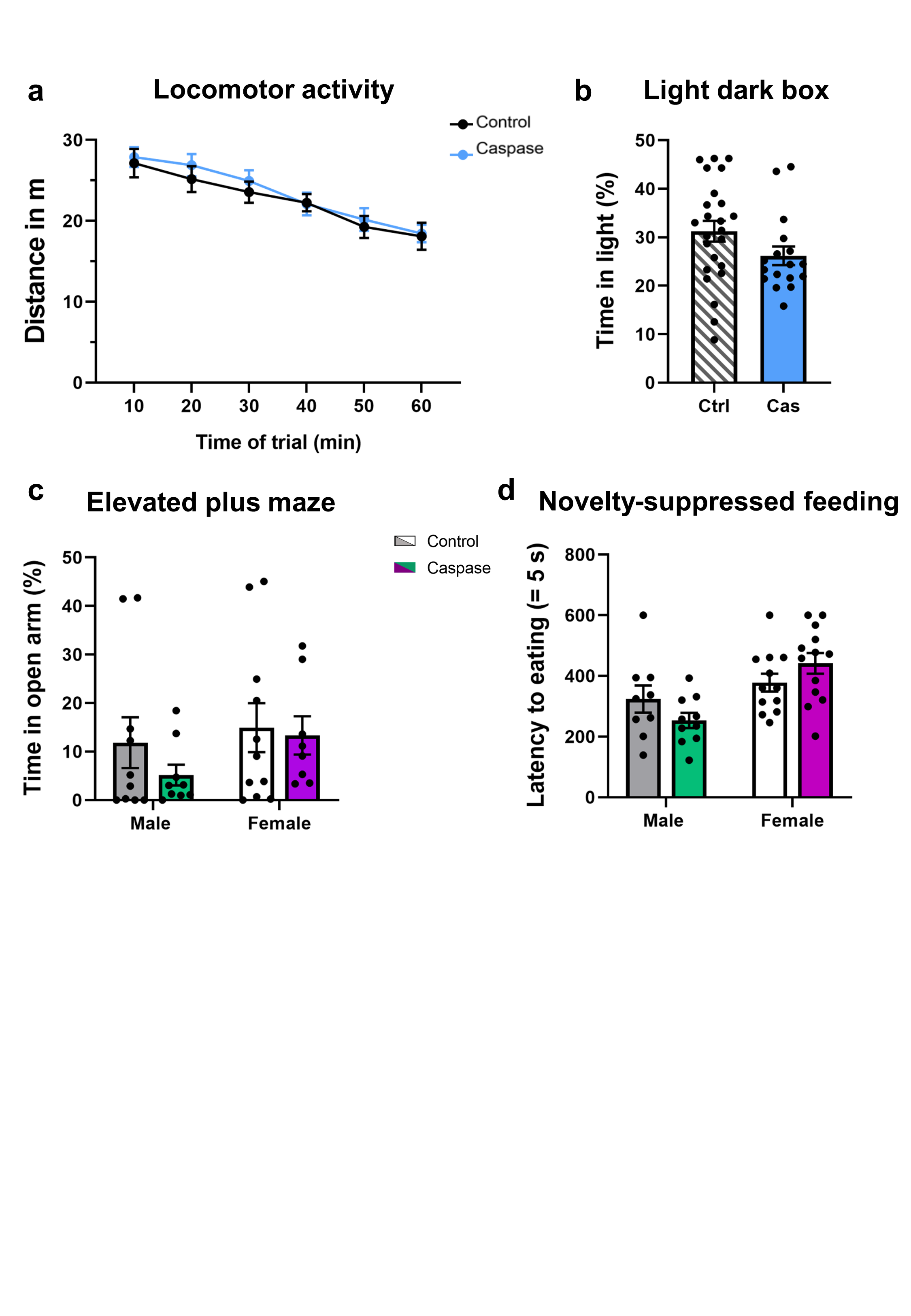

Supplement: Figure 5-2 — Deletion of Sst+ neurons in the VTA had no effect on locomotor activity or anxiety-like behavior. a, Locomotor activity in the open arena was not different between the VTASst-caspase and control mice (F(1,34) = 0.265, p = 0.61). b, The light/dark box test did not show any difference in percentage of time spent in the light compartment between the treatment groups (F(1,34) = 1.750, p = 0.195; sex, F(1,34) = 3.957, p = 0.055). c, Similarly, the percentage of time spent in the open arm measured in the elevated plus maze test was not different (treatment, F(1,34) = 0.072, p = 0.79). d, Latency to start eating in a novel environment did not show a difference, albeit a marginal significance for sex-dependent effects was detected in the VTASst-caspase mice (treatment × sex, F(1,34) = 3.862, p = 0.058). Data are shown as mean ± SEM. Download Figure 5-2, TIF file. [file enu-eN-NWR-0149-23-s08.tif]

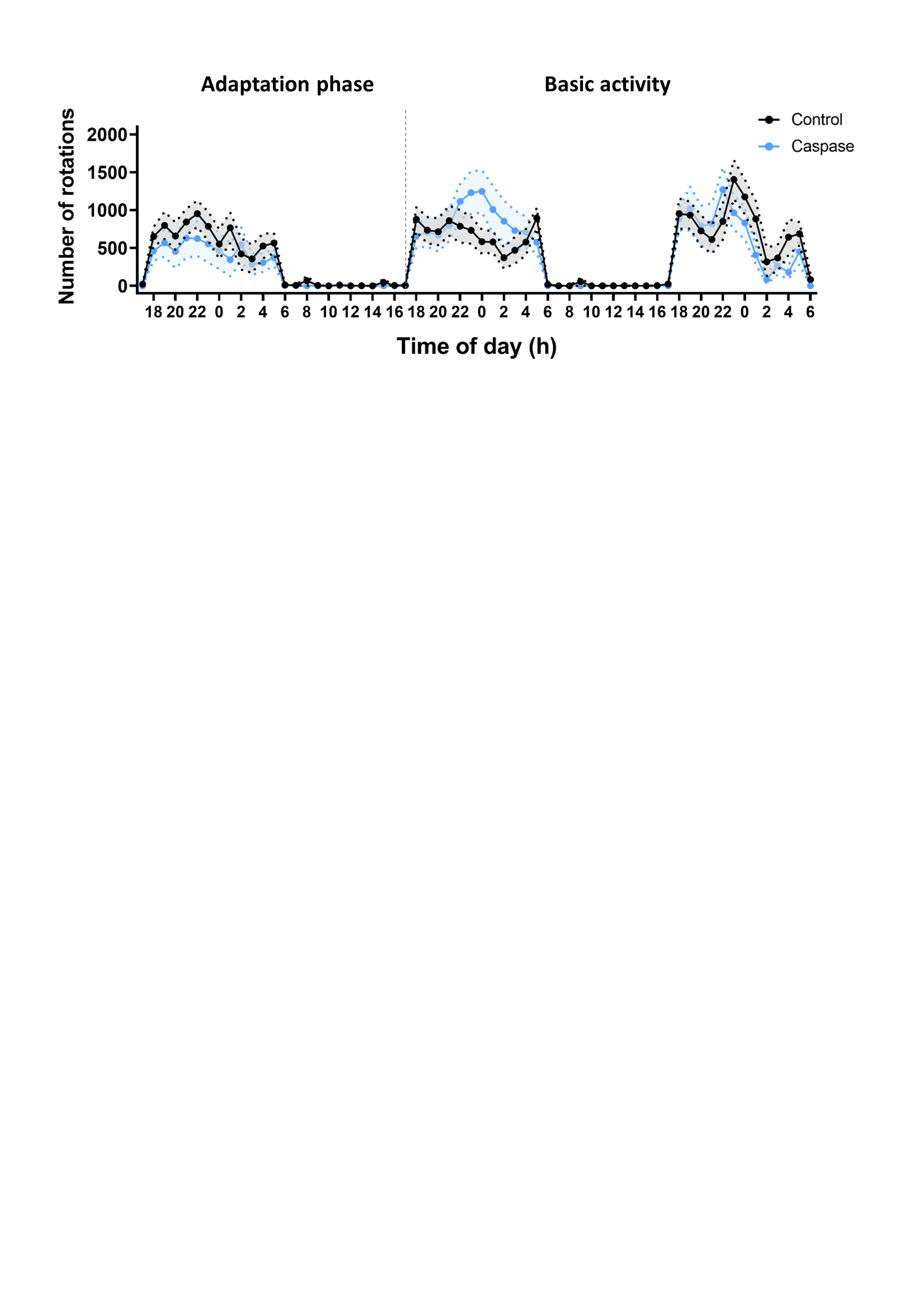

Supplement: Figure 5-3 — Deletion of VTASst neurons did not affect circadian activity in the free-running wheel test (lights on 6:00–18:00). Activity of the control (black) and VTASst-caspase (blue) mice. There were no differences in the number of rotations between the treatment groups across 3 days (treatment, F(1,24) = 0.202, p = 0.657; treatment × time, F(67,1608) = 1.230, p = 0.278). Data are shown as mean ± SEM. Download Figure 5-3, TIF file. [file enu-eN-NWR-0149-23-s09.tif]

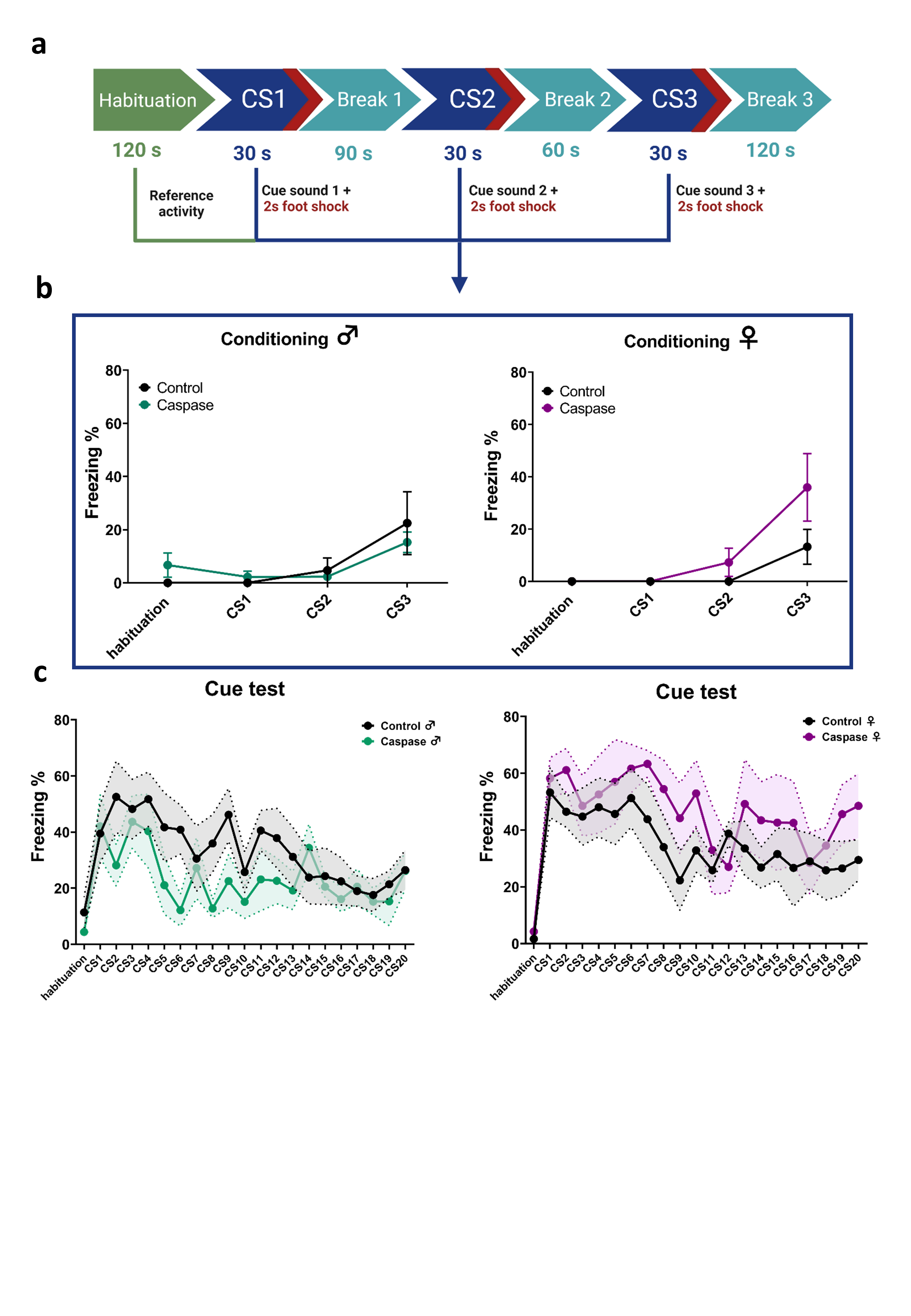

Supplement: Figure 6-1 — Deletion of the VTASst neurons did not affect cue-induced fear processing in pavlovian fear conditioning. a, Protocol of fear conditioning during the acquisition phase. b, Graphs show percentage of freezing (freeze time/total time) during 30 s cue-sound presentations, coterminated with 2 s footshocks. There was no difference in freezing between sexes (F(1,19) = 0.019, p = 0.891) or between treatments (F(1,19) = 2.182, p = 0.156) . c, Similarly, there were no significant differences in rates of cue-associated fear memory retrieval or extinction (cue × sex × treatment, F(1,420) = 1.04, p = 0.413). Data are shown as mean ± SEM. Download Figure 6-1, TIF file. [file enu-eN-NWR-0149-23-s10.tif]

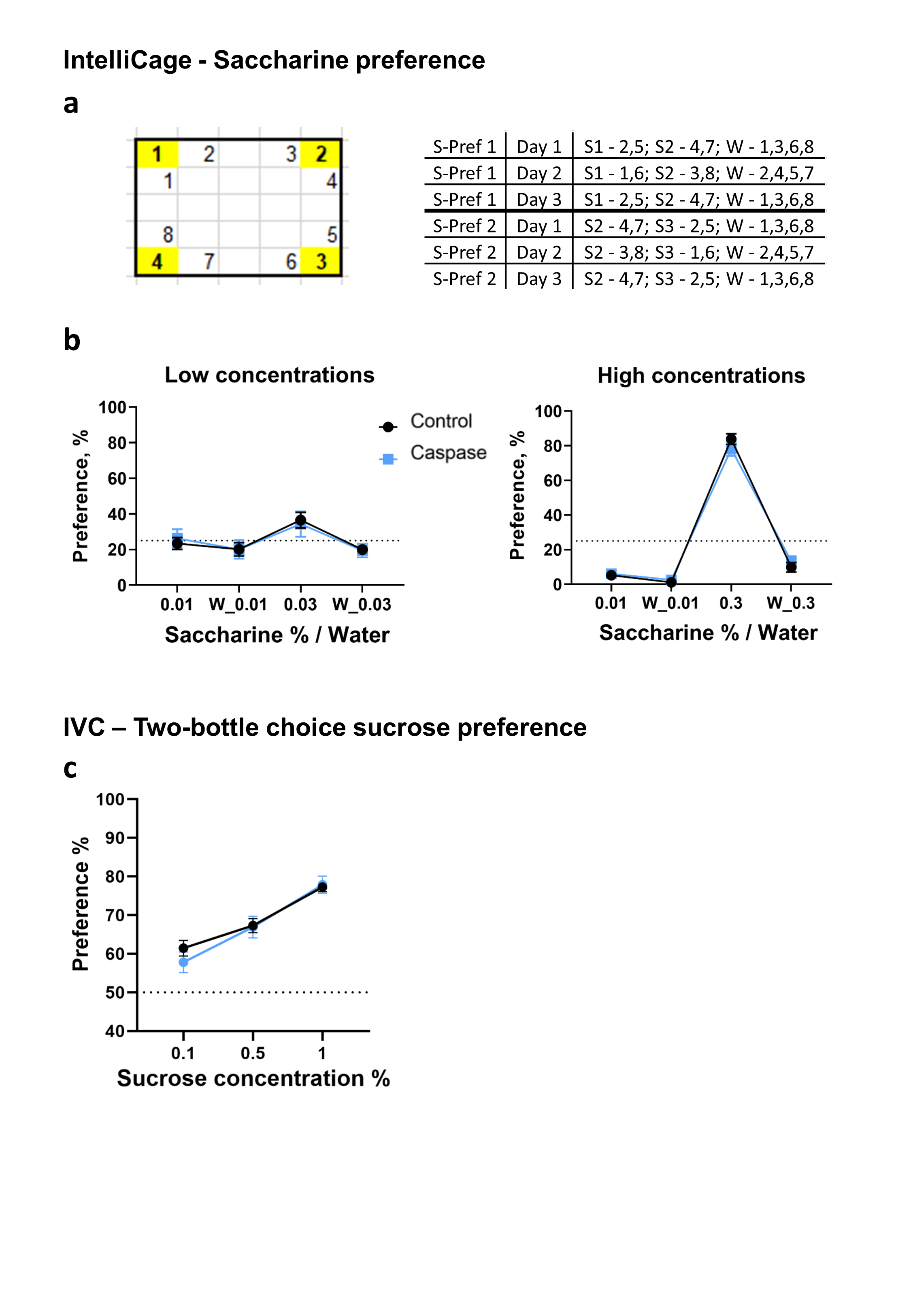

Supplement: Figure 8-1 — Deletion of the VTASst neurons did not affect natural reward preference or sensitivity. a, Simplified scheme depicting corners (yellow) and side assignment of the saccharine preference test in the IntelliCage system. S1, 0.01% saccharine; S2, 0.03% saccharine; S3, 0.3% saccharine; W, water. b, Graphs show preference in percentages (number of licks to a certain bottle/number of total licks, y-axis) to different saccharine concentrations over water (x-axis). Preference to different saccharine concentrations or water in the corresponding corner in the IntelliCage system did not reveal any significant differences between the treatment groups for low saccharine concentrations (F(1,24) = 0.698, p = 0.413) or to high ones (F(1,24) = 0.045, p = 0.834). The dashed line shows a 25% preference rate. c, Similarly, sucrose preference in the two-bottle choice test in IVC cages did not reveal any differences (concentration × treatment, F(2,66) = 0.362, p = 0.688). The dashed line shows a 50% preference rate. Data are shown as mean ± SEM. Download Figure 8-1, TIF file. [file enu-eN-NWR-0149-23-s11.tif]

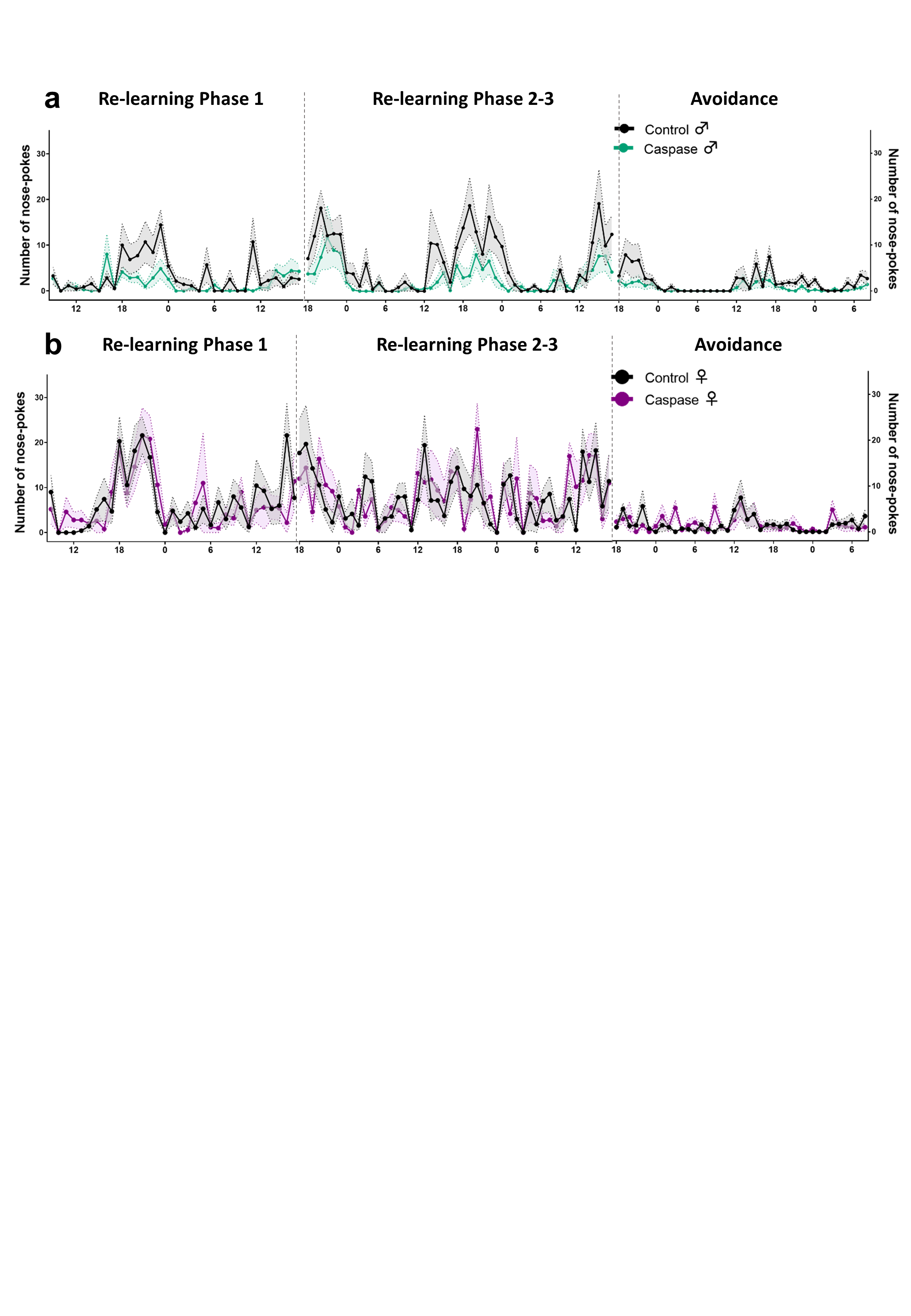

Supplement: Figure 8-2 — Relearning new rules in control and caspase mice and introduction of air puffs in the reward-related task. The x-axis shows the number of nose pokes to the saccharine bottles per hour, y-axis shows daily hours (lights on 6:00–18:00). a, Nose-poking dynamics in male mice. Although there was a clear tendency in VTASst-caspase male mice to be less active in nose poking to the saccharine corner in all phases of the relearning-avoidance test, no statistically significant differences were detected between the groups (Extended Data Table 5-1). b, Nose-poking dynamics in female mice showed no differences between the groups. Data are shown as mean ± SEM. Download Figure 8-2, TIF file. [file enu-eN-NWR-0149-23-s12.tif]
